# Self-assembled skin-like metamaterials for dual-band camouflage

**DOI:** 10.1126/sciadv.adl1896

**Published:** 2024-06-19

**Authors:** Shiqi Fang, Ning Xu, Lin Zhou, Tianqi Wei, Yuhan Yang, Yongmin Liu, Jia Zhu

**Affiliations:** ^1^ National Laboratory of Solid State Microstructures, College of Engineering and Applied Sciences, Jiangsu Key Laboratory of Artificial Functional Materials, Frontiers Science Center for Critical Earth Material Cycling, Collaborative Innovation Center of Advanced Microstructures, Nanjing University, Nanjing 210093, China.; ^2^ Department of Mechanical and Industrial Engineering, Northeastern University, Boston, MA 02115, USA.; ^3^ School of Sustainable Energy and Resources, Nanjing University, Suzhou 215010, China.

## Abstract

Skin-like soft optical metamaterials with broadband modulation have been long pursued for practical applications, such as cloaking and camouflage. Here, we propose a skin-like metamaterial for dual-band camouflage based on unique Au nanoparticles assembled hollow pillars (NPAHP), which are implemented by the bottom-up template-assisted self-assembly processes. This dual-band camouflage realizes simultaneously high visible absorptivity (~0.947) and low infrared emissivity (~0.074/0.045 for mid-/long-wavelength infrared bands), ideal for visible and infrared dual-band camouflage at night or in outer space. In addition, this self-assembled metamaterial, with a micrometer thickness and periodic through-holes, demonstrates superior skin-like attachability and permeability, allowing close attachment to a wide range of surfaces including the human body. Last but not least, benefiting from the extremely low infrared emissivity, the skin-like metamaterial exhibits excellent high-temperature camouflage performance, with radiation temperature reduction from 678 to 353 kelvin. This work provides a new paradigm for skin-like metamaterials with flexible multiband modulation for multiple application scenarios.

## Teaser

A skin-like metamaterial with a highly selective spectrum is designed and fabricated for dual-band compatible camouflage.

## INTRODUCTION

Optical metamaterials, artificially structured materials consisting of subwavelength meta-atoms for fulfilling the prescribed properties (*1*–*3*), hold great promise for various applications such as cloaking, imaging, and camouflage (*4*–*9*). The construction of meta-atoms at a deep subwavelength scale is intrinsically challenging and typically requires complicated fabrication techniques (*10*). Advanced top-down lithograph processes, such as focused ion beam (FIB) (*11*–*14*) and electron beam lithography (EBL) (*15*–*19*), have been developed for fabricating extremely fine patterns in nanoscale. Bottom-up approaches are exploited to construct desired architectures based on self-assembly with potentially lower cost (*20*–*22*) while being applicable to strict material constraints and taking lots of effort for structure control. Thus, for practical applications, such as personal thermal management (*23*–*26*), smart window (*27*–*30*), photothermal catalysis (*31*–*33*), and multispectral camouflage (*34*–*41*), which need broadband modulation and large-scale production, it poses further requirements on material designs and processes.

Here, taking camouflage as an example, with the perpetual advancements in various detection approaches at different wavelengths, it is crucial to regulate optical properties over broadband. Camouflage at night or in outer space is a common application scenario, especially in the military field, which requires both high visible absorptivity to fit the black background (*42*, *43*) and low infrared emissivity to suppress radiation at the same time (*44*, *45*). Furthermore, some harsh but crucial application scenarios, such as disguising living bodies (*46*) or high-temperature objects (*47*–*49*), have higher requirements on material/structure design. Specifically, when camouflage materials are used for soldiers performing military missions, permeability and softness are required to ensure wear comfort (*50*, *51*). Another urgent but challenging issue for the military is the infrared camouflage of high-temperature objects. According to Stefan-Boltzmann law, *P* = εδ*T*^4^, extremely low emissivity is pursued for excellent infrared camouflage. On the basis of all the requirements, it is desired to design and fabricate a skin-like soft optical metamaterial with a highly selective spectrum, i.e., high absorptivity (α > 0.9) in the visible band (VIS: 380 to 780 nm) and extremely low emissivity (ε < 0.1) in the mid- and long-wavelength infrared bands (MWIR: 3 to 5 μm, LWIR: 8 to 14 μm).

However, it is challenging for traditional materials to meet the selective-spectrum requirements of the compatible camouflage. For instance, they usually have high reflection [metallic materials (*1*)] or high absorption [carbon-based materials (*52*, *53*)] over a broad spectrum (VIS-LWIR). In recent years, many efforts have been devoted to developing artificial materials for compatible camouflage, such as metasurfaces, multilayer films, and MXene. Metasurface-based camouflage materials with top-down lithograph processes can enable finely tuning optical properties (*37*, *40*, *54*, *55*). However, they often come with extensive fabrication costs. Multilayer films achieve flexible spectral modulation by precisely designing the material and thickness of different layers (*35*, *38*, *56*–*58*), which, in turn, requires a more complex fabrication process, leading to low structural tolerance. Besides, thermal mismatch of the multilayer film is also a concern at high working temperatures (*59*, *60*). Moreover, considering the reliance on the substrate for metasurfaces and multilayer films, ensuring their attachability and permeability is usually challenging. MXene is proposed as another emerging camouflage material due to its intrinsic selective spectrum with average visible absorptivity (α) of ~0.8 to 0.91 and average infrared emissivity (ε) of ~0.06 to 0.3 in the MWIR/LWIR bands (*43*, *49*, *61*). This makes it promising for visible and infrared compatible camouflage in dark environments (at night or in outer space). Meanwhile, it is worthwhile to explore other material systems toward higher spectral selectivity as well as other favorable characteristics, such as a simple fabrication process, great attachability, and permeability. These diverse material systems will provide more options for various camouflage scenarios, promoting the advancement of the camouflage technology.

**Fig. 1.**
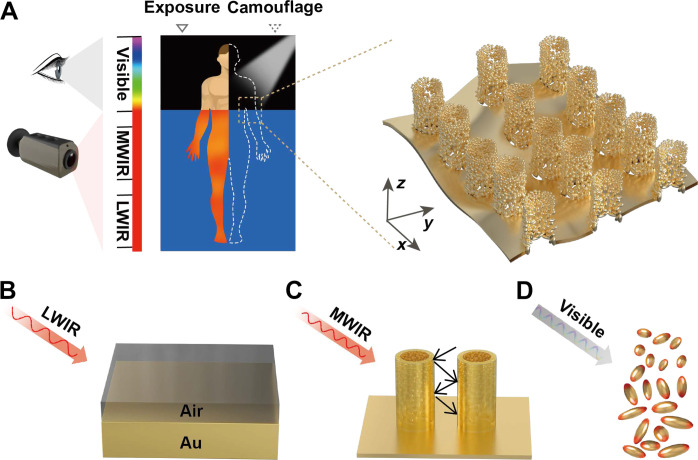
Schematic and mechanism of the NPAHP-based skin-like metamaterial for dual-band camouflage. (**A**) Schematic of the NPAHP-based skin-like metamaterial attached to the human body surface (exposure: uncovered part; camouflage: covered with NPAHP-based skin-like metamaterial) for the visible and infrared dual-band camouflage. (**B**) For the LWIR band, nanoscale structures are negligible, and the optical property is mainly dependent on the filling ratio of Au (*f*) by effective medium theory. (**C**) For the MWIR band, the multiple scattering effect of Au pillars (hundreds of nanometers in scale) dominates the optical property. (**D**) For the visible band, the LSPR effect of Au NPs (tens of nanometers in scale) plays the dominant role.

In this work, we fabricate a skin-like metamaterial with a highly selective spectrum for dual-band camouflage based on unique Au nanoparticles assembled hollow pillars (NPAHP) (Fig. 1A). By using a bottom-up template-assisted self-assembled strategy, Au nanoparticles (NPs) (tens of nanometers in scale) are deposited and bond together to form microscale hollow pillars (hundreds of nanometers in scale) and are vertically arranged on macroscale Au film with periodic through-holes. This cross-scale three-dimensional hierarchical structure enables tailored optical properties. Specifically, for the LWIR band, nanoscale structures (Au NPs and pillars) are negligible, and the optical property is dependent on the overall filling ratio of Au (*f*) based on the effective medium theory (Fig. 1B). While for the MWIR and visible bands, the absorption performance is respectively dominated by the multiple scattering of the subwavelength hollow pillars (Fig. 1C) and the localized surface plasmon resonance (LSPR) hybridization effect of Au NPs (Fig. 1D) (*62*, *63*). Thus, by fine-tuning the hierarchical structure, this NPAHP-based metamaterial is capable of selectively enhancing visible absorptivity (~0.947 from 0.38 to 0.78 μm) while simultaneously maintaining low infrared emissivity (~0.074/0.045 for the MWIR/LWIR bands), achieving effective visible and infrared camouflage at night or in outer space.

Meanwhile, the NPAHP-based metamaterial has a thickness of ~0.6 μm and periodic through-holes, which endow it with skin-like attachability and permeability, allowing it closely attached to almost arbitrary surfaces including the human body. Besides, taking advantage of extremely low infrared emissivity, NPAHP-based skin-like metamaterial (with 10-nm SnO_2_ coating for protection in high-temperature conditions) exhibits excellent high-temperature camouflage performance, with radiation temperature reduction from 678 to 353 K. These comprehensive properties make the NPAHP-based film a promising candidate for camouflage materials (table S1).

## RESULTS

### Structural design of the NPAHP-based skin-like metamaterial with an ideally selective spectrum

To achieve the dual-band camouflage, we first numerically study the effect of the structural design of the NPAHP-based film on the absorption spectrum by the finite-different time-domain method (detailed simulated information is present in note S1). As absorption performance for different bands is dominated by the structures with three different scales (that is, particles with tens of nanometers in scale, pillars with hundreds of nanometers in scale, and film in macroscale), three simplified optical models are adopted.

**Fig. 2.**
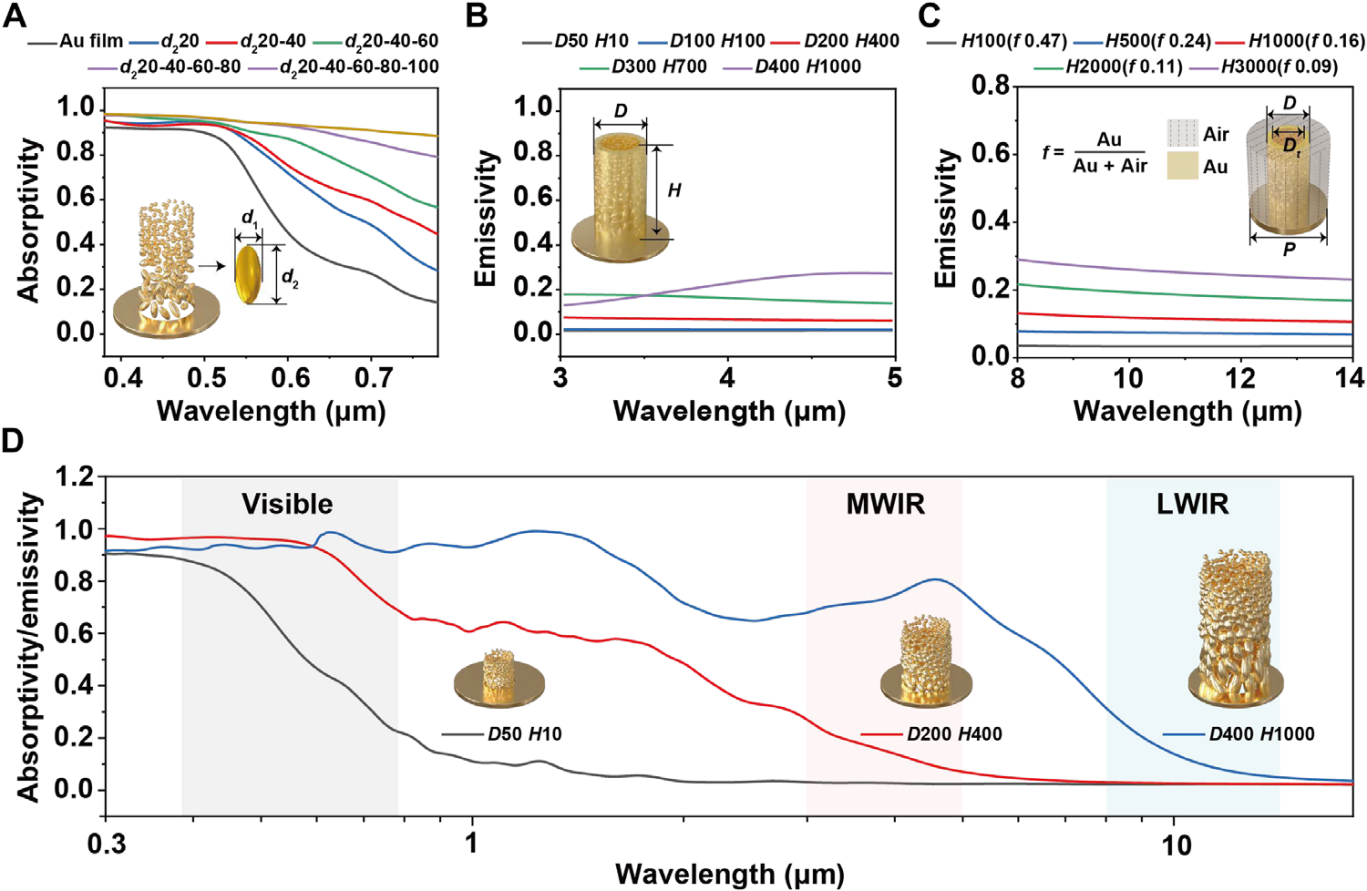
Simulated absorptivity/emissivity spectra of the NPAHP-based skin-like metamaterial with different structural designs. (**A**) Dependence of the absorptivity/emissivity on the Au NP size distribution in the visible band. (**B**) Dependence of the absorptivity/emissivity on the *D* and *H* of Au hollow pillars in the MWIR band. (**C**) Dependence of the absorptivity/emissivity on the Au ratio (*f*) in the LWIR band. (**D**) Full-wave electromagnetic simulation for three representative structure models over 0.3 to 18 μm.

For enhancing the absorption performance of NPAHP-based film in the visible band, the size distribution of the Au NPs needs to be well studied and tailored. This is because Au NPs with tens of nanometers in scale strongly interact with visible light due to the collective oscillation of conductive electrons excited by electromagnetic waves, namely, the LSPR effect. As particle size increases, the resonance absorption peak of the particle will red-shift (fig. S1). Thus, particles with diverse sizes will induce the LSPR hybridization effect, leading to multiple overlapping plasmonic modes. Therefore, the dependence of absorption performance in the visible band on particle size distribution indicates that the absorption band can be broadened when particles have wider particle size distribution. As shown in Fig. 2A, single-sized particles have an absorption cutoff at 520 nm, while multisized particles demonstrate high absorption over the entire visible spectrum.

To suppress the emissivity in the MWIR band, the structure of the particle-assembled pillar should be elaborately controlled, as the absorptivity/emissivity in the MWIR band is sensitive to the structure with hundreds of nanometers in scale, despite the pillar structure helps further enhance visible absorptivity by light scattering (fig. S3). The effect of the pillar geometry, including diameter *D* and height *H*, for absorptivity/emissivity in MWIR is studied in Fig. 2B. Here, Au NPs can be neglected since NPs with tens of nanometers in diameter hardly affect the absorptivity in MWIR band. The optical simulation model is simplified to a three-dimensional hollow Au pillar with a thin and uniform sidewall. It is indicated that as the geometry (*D*, *H*) of the pillars gradually approaches the subwavelength scale, its effect on the absorption performance in the MWIR band becomes increasingly significant.

For maintaining the low emissivity in the LWIR band, the Au ratio of the film counts and should be well studied. The NPAHP-based film can be regarded as a homogeneous medium filling of Au and air, as both Au NPs and pillars are negligible due to their extremely small scales compared to the wavelength. Thus, the effective medium theory can be used for the simulation of the absorption spectrum in the LWIR band. In Fig. 2C, we studied the dependence of emissivity on different filling ratios (*f*) of Au (*f* varies with *H*) for 8 to 14 μm. It is observed that the reduced Au ratio enhances the emissivity in the LWIR band.

After figuring out the key factors for the concerned bands, full-wave electromagnetic simulations are also carried out on the basis of three representative structure models as shown in Fig. 2D, given the fabrication method (specific details are found in note S1). All the above simulations suggest that the design of the NPAHP-based hierarchical structure is capable of achieving synergistic modulation in the visible, MWIR, and LWIR bands based on the LSPR hybridization effect of Au NPs, multiple scattering of subwavelength-sized pillars, and effective medium theory in the macroscopic system, respectively. Optimizing structural parameters (middle-size NPAHP) can enable the highly selective spectrum, i.e., high absorptivity in the visible band and low emissivity in the MWIR/LWIR bands, which is favorable for the effective visible (at night or in outer space) and infrared dual-band compatible camouflage.

### Fabrication and characterization of the NPAHP-based skin-like metamaterial

**Fig. 3.**
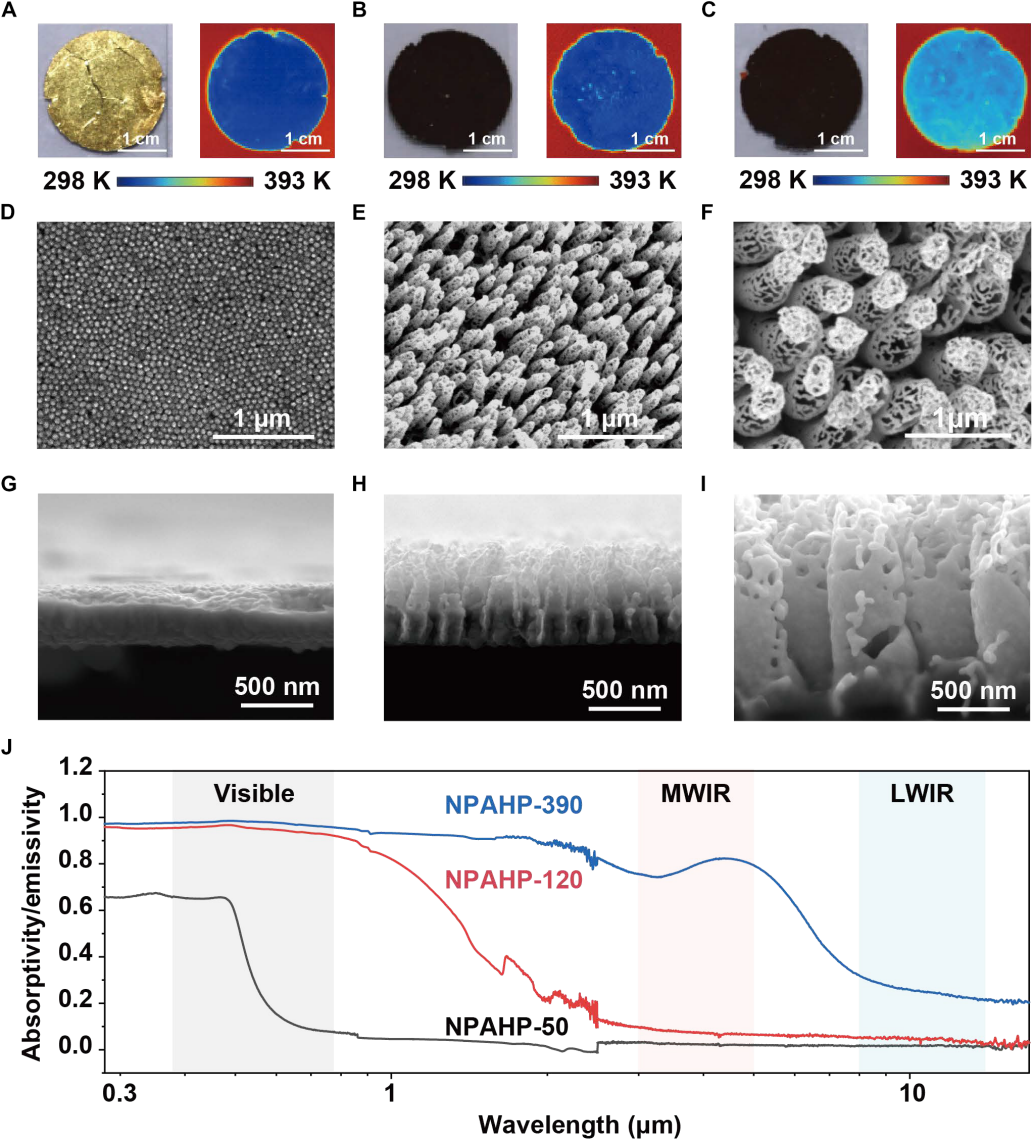
Fabrication and characterization of the NPAHP-based skin-like metamaterial. (**A** to **C**) Optical photographs (left) and infrared photographs (right) for NPAHP-50 (A), NPAHP-120 (B), and NPAHP-390 (C). (**D** to **F**) Top view images of SEM for NPAHP-50 (D), NPAHP-120 (E), and NPAHP-390 (F). (**G** to **I**) Cross-sectional view images of SEM for NPAHP-50 (G), NPAHP-120 (H), and NPAHP-390 (I). (**J**) Experimental absorption spectra for NPHAP-50/120/390 show that NPAHP-120 exhibits the best dual-band camouflage performance.

Experimentally, the desired NPAHP-based skin-like metamaterial was fabricated by a simple two-step template method (notes S2 to S6), including (i) depositing Au NPs on anodic aluminum oxide (AAO) nanoporous template by e-beam evaporation and (ii) etching AAO template in NaOH solution while retaining the Au-based structure (fig. S1). In this process, the pore size (*D*) of the AAO template plays a crucial role in manipulating nanostructures, as structures (*D* and *H* of the hollow pillars and size distribution of particles) of NPAHP vary when using different AAO templates with different *D*. Thus, we obtained three samples (marked as NPAHP-50/120/390) based on the AAO template with different pore sizes (*D* = 50, 120, and 390 nm, marked as AAO-50/120/390). It is observed that NPAHP-50 appears golden (Fig. 3A), and NPAHP-120 and NPAHP-390 present black (Fig. 3, B and C). It is worth mentioning that the fabrication of NPAHP structures exhibits excellent controllability based on the AAO template method, which exhibits highly consistent spectra of NPAHP-50/120/390 across multiple experiments (note S7).

To characterize the micromorphology of three samples, the scanning electron microscopy (SEM) images of the top view and cross-sectional view are shown in Fig. 3 (D to I). Different from the simulated ideal structure with periodic through-holes in Fig. 2D, because of the extremely small pore size of AAO-50, the Au NPs cannot be deposited into the holes, making the etched sample (NPAHP-50) appears as an Au film with periodic slight protrusions (without through-holes) (Fig. 3, D and G). Thus, it presents the lowest infrared emissivity (similar to bulk Au film) and poorly visible absorptivity (~0.316) (Fig. 3J). The simulated absorption spectra based on the experimental structures are also presented in note S9.

When using the template of AAO-390, owing to the large pore size and porosity, a considerable portion of the evaporated Au clusters penetrate into the nanopores, colliding, aggregating, and eventually adhering on the side walls, forming closely packed NPs with wide size distribution. Therefore, during the etching, most of the Au NPs are retained by the physical adhesion between particles, forming the self-supporting Au pillars (*D* ~ 390 nm, *H* ~ 1 μm) (Fig. 3, E and H). Benefiting from the wide particle size distribution, high absorptivity (~0.974) over 0.38 to 0.78 μm is achieved in NPAHP-390. However, the large *D* and *H* of the pillars and low Au ratio lead to higher infrared emissivity (~0.795/~0.252 for the MWIR/LWIR bands) (Fig. 3J).

For the intermediate state (AAO-120), the number of Au NPs distributed in nanopores is less, and the penetration depth of Au NPs inside nanopores is shallower due to the relatively small pore size compared to AAO-390. Thus, the etched sample (NPAHP-120) remains smaller Au pillars (*D* ~ 120 nm, *H* ~ 300 nm) but still with wide particle distribution (Fig. 3, F and I), which shows the most similarity to the ideal structural model simulated in Fig. 2D. Therefore, the NPAHP-120 exhibits the desired selective spectrum, i.e., high absorptivity (~0.947) in the visible band and low emissivity (~0.074/~0.045) in the MWIR/LWIR bands (Fig. 3J). In addition, the NPAHP-120 exhibits excellent angle insensitivity over a wide incident angle (0° to 50°) in both visible and infrared bands (note S8).

The above structural and spectral properties are consistent with the optical and infrared photographs of NPAHP-50/120/390 (Fig. 3, A to C). The visually golden NPAHP-50 and the relatively high radiation temperature of NPAHP-390 hinder their application for dual-band compatible camouflage, respectively (Fig. 3, A and C). Different from NPAHP-50/390, the NPAHP-120 presents black visually while having low radiation temperature due to the ideal structure (Fig. 3B), having excellent dual-band camouflage performance.

### NPAHP-120–based skin-like metamaterial for dual-band camouflage

**Fig. 4.**
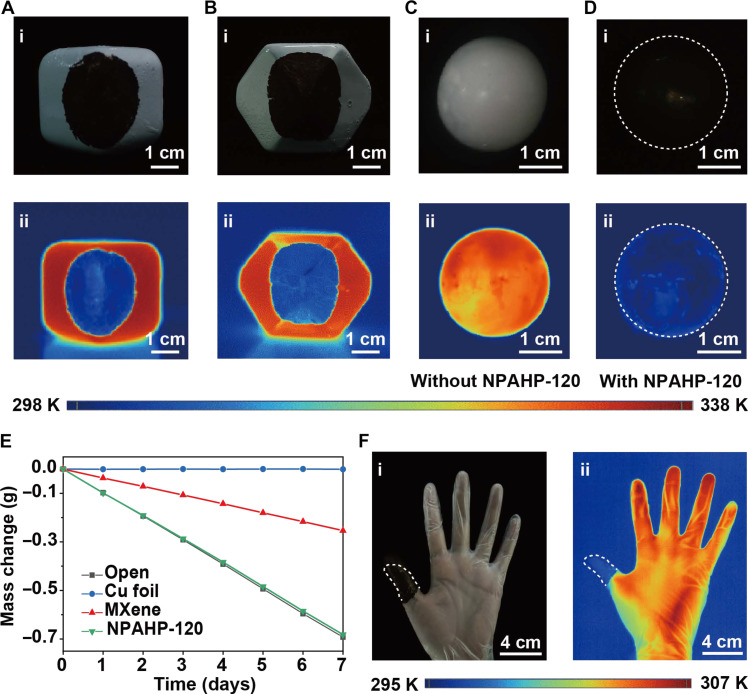
NPAHP-120–based skin-like metamaterial for dual-band camouflage, demonstrating good attachability and wearable potential. (**A** and **B**) Optical and infrared photographs of the NPAHP-120–based film attached to painted stainless steel polyhedra. (**C** and **D**) Optical and infrared photographs of a spherical object uncovered (C)/covered (D) with the NPAHP-120–based film. (**E**) Water vapor transmission tests of the open state, the NPAHP-120–based film, MXene, and Cu foil. (**F**) Optical and infrared photographs of the NPAHP-120–based film attached to a finger.

In addition to the selective optical spectrum, the NPAHP-120–based skin-like metamaterial presents skin-like attachability and permeability, desirable for the actual application of camouflage. Owing to the ultrathin thickness (~0.6 μm) of the film, it exhibits great attachability to arbitrary surfaces. As shown in Fig. 4 (A and B), it displays that the NPAHP-120–based film can be closely attached to the painted stainless steel polyhedra, even the angular areas of them. The attached areas thus are well concealed from visible and infrared detection. Furthermore, the camouflage film can completely encase a spherical object, making it almost perfectly hidden in the surroundings, when detected by either visible or infrared detectors (Fig. 4, C and D). Besides, the close contact and the Van der Waals between the NPAHP film and the substrate provide the NPAHP film good adhesiveness to the substrate. In note S10, we characterize that the sample will also not detach from the glass substrate at different tilt angles or even when completely inversed (fig. S14A). Furthermore, for simulating the tougher condition, the wind speed approaching 3.0 m/s is introduced, it is observed that the sample still does not detach from the substrate (fig. S14B).

Besides, with the assistance of the nanoporous AAO template, the etched sample, NPAHP-120, is endowed with great permeability due to its periodic through-holes. Water evaporation tests were performed to quantitatively measure its permeability. Water evaporation is hardly affected when covered with the NPAHP-based metamaterial (Fig. 4E). In contrast, water evaporation is seriously suppressed when covered with conventional camouflage films (MXene and Cu foil). These results show that NPAHP-based metamaterial has excellent vapor permeability, much better than that of conventional camouflage materials. Besides, the air permeability test also demonstrates that the NPAHP-120 film exhibits similar great air permeability as commercial cotton in note S11 (fig. S15). Together with great attachability, the NPAHP-based skin-like metamaterial is promising to be an appealing alternative in the wearable camouflage field. As shown in Fig. 4F, the NPAHP-based skin-like metamaterial is capable of being tightly attached to the human body. These unique features have seldom been reported for conventional camouflage materials but are important for various camouflage scenarios. In addition, the almost unchanged spectra of NPAHP-120 film after being placed in different environmental temperature and humidity conditions over 1 month (fig. S16) indicate its optical stability (note S12). Furthermore, the NPAHP-120 film also demonstrates stable optical performance even after being folded over 100 times for simulating the repeated usage conditions (note S12). As shown in fig. S17, the optical photographs and spectra of our NPAHP-120 film remain almost the same before and after folding, indicating the durability of our NPAHP-120 film.

### High-temperature camouflage performance of NPAHP-120–based skin-like metamaterial

In addition to the excellent wearable camouflage performance of NPAHP-120–based skin-like metamaterial in living bodies, it is also capable of maintaining its good camouflage performance even when attached to high-temperature objects, owing to its ultralow infrared emissivity. Here, we first quantitatively calculate the effect of infrared emissivity on camouflage performance with increasing temperature (notes S13 and S14). The camouflage performance can be evaluated based on the power (or temperature) detected by an infrared camera. The detected power (*P*_det_) is composed of two parts: (i) the radiative power from the object (*P*_rad_) and the reflective power of ambient radiation by the object (*P*_ref_).

$$P_{\text{det}}\left(\varepsilon_{\text{IR}},T_{r}\right)=P_{\text{rad}}\left(\varepsilon_{1},T_{1}\right)+P_{\text{ref}}\left(\varepsilon_{1},\varepsilon_{a},T_{a}\right)$$ (1)

where ε_IR_ is the emissivity set on the infrared camera; *T*_r_ is the detected radiation temperature of the object; ε_1_ and ε_a_ are the emissivity of the object and surroundings, respectively; *T*_1_ and *T*_a_ are the actual temperatures of the object and surroundings, respectively. For most natural environments, the ambient emissivity is close to that of a blackbody; thus, ε_a_ and *T*_a_ are set to be 1.0 and 298 K, respectively.

**Fig. 5.**
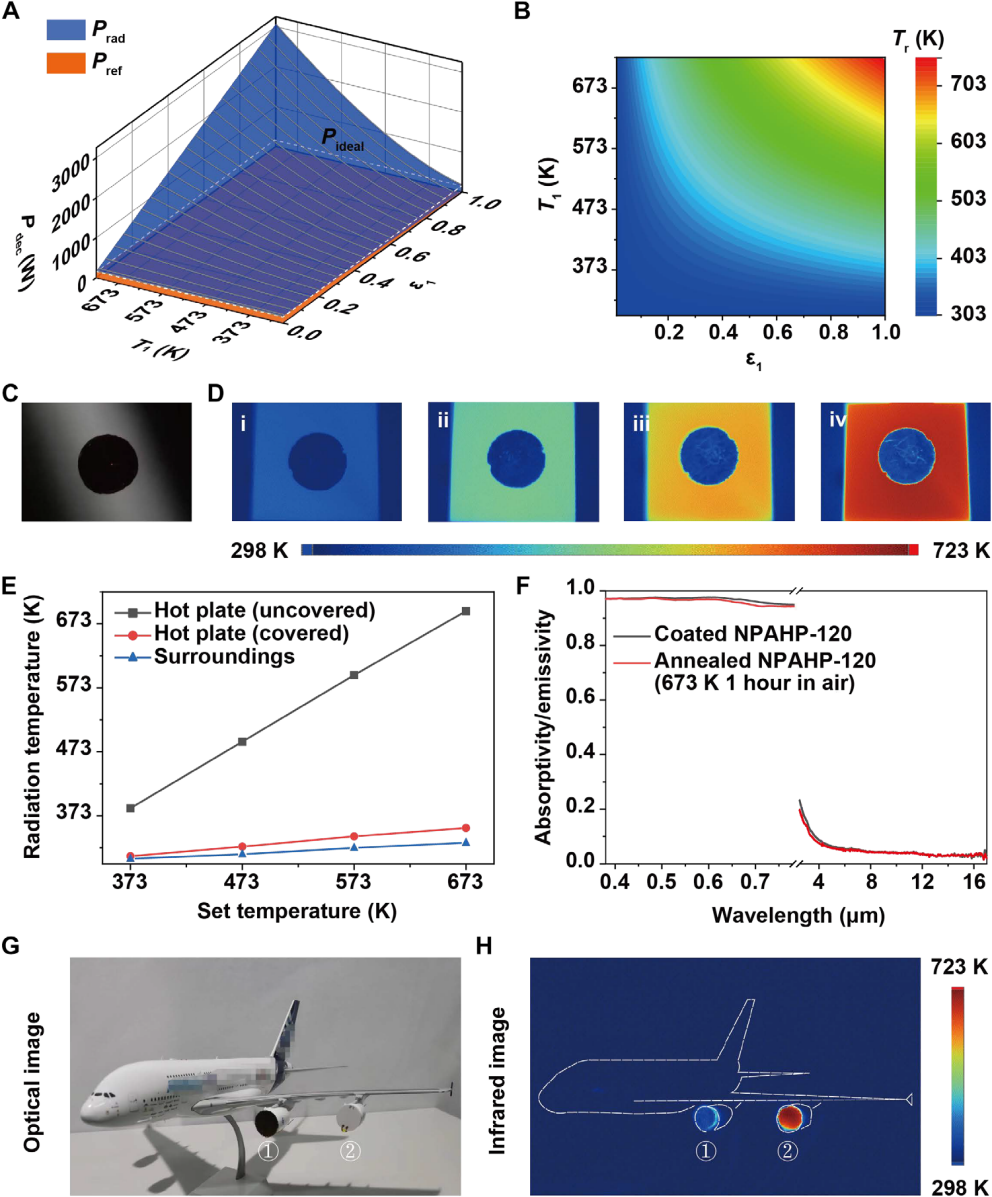
Demonstration of high-temperature camouflage performance of the NPAHP-120–based skin-like metamaterial. (**A** and **B**) Dependence of *P*_det_ (A) and *T_r_* (B) on *T*_1_ and ε_1_. (**C**) Optical photograph of the NPAHP-120–based camouflage film. (**D**) Infrared photographs of the hot plate covered with the NPAHP-120–based camouflage film at different set temperatures (373, 473, 573, and 673 K). (**E**) Detected radiation temperature of the hot plate (covered and uncovered with NPAHP-120–based film) and surroundings at different temperatures. (**F**) The unchanged experimental absorption spectra of the NPAHP-120–based camouflage film after air annealing at 673 K for 1 hour, which suggests its good stability at high temperature. (**G** and **H**) Optical (G) and infrared photograph (H) of the aircraft engine models covered (①) and uncovered (②) with the NPAHP-120–based camouflage film.

As shown in Fig. 5A, the dependence of *P*_det_, *P*_rad_, and *P*_ref_ on *T*_1_ and ε_1_ are illustrated, respectively. Here, the dashed line represents the ideal state *P*_ideal_, i.e., the object is completely integrated with its surroundings. Obviously, in most cases, *P*_det_ is higher than *P*_ideal_ and increases exponentially with *T*_1_, while for the object with low emissivity, the *P*_det_ is much less than that of a blackbody, especially at high temperatures. Furthermore, the dependence of *T*_r_ on *T*_1_ and ε_1_ intuitively indicates that for every 0.01 reduction in ε_1_, the *T*_1_ is allowed to greatly increase under the premise of the isoradiation temperature, especially in low emissivity regions (<0.1) (Fig. 5B). The simulation results suggest that extremely low emissivity is of great importance for high-temperature camouflage.

The experiments also demonstrate the excellent dual-band camouflage performance when treating high-temperature objects. The visually black NPAHP-120–based film (coated with 10-nm SnO_2_; note S15) (Fig. 5C) is covered on the hot plate with different set temperatures (373, 473, 573, and 673 K), and the infrared photographs are shown in Fig. 5D. The specific radiation temperature of the hot plate (covered and uncovered with NPAHP-120) and surroundings are recorded in Fig. 5E. It shows that the detected temperatures are highly suppressed after covering with the NPAHP-120–based film (hot plate of ~678 K can be detected as 353 K) and nearly the same with that of the surroundings, indicating excellent high-temperature camouflage performance of NPAHP-120–based film. We also confirm the high-temperature stability of the camouflage film. The optical spectra and structures remain almost unchanged after air annealing at 673 K for 1 hour in Fig. 5F and fig. S19 (note S16). It is worth noting that, although the high visible absorptivity and low infrared emissivity achieved by this metal NP-based structure are attractive in the field of camouflage, its long-term stability at high temperatures is indeed a challenge. It is expected that the high-temperature stability of the NPAHP structure will be further improved if it can be coated with a more stable and dense oxide material (such as HfO_2_) via the atomic layer deposition (ALD) method. In addition, efficient thermal management, such as introducing thermal insulation aerogel to reduce the actual temperature of camouflage materials, is considered an effective strategy to further improve the high-temperature camouflage performance of our NPAHP-120 film.

The excellent high-temperature camouflage performance of the NPAHP-120–based film greatly widens its application scenarios. For example, the camouflage material is attached to the aircraft engine model in Fig. 5 (G and H). It is obvious that once covered with the NPAHP-120–based film (the position marked with ①), it presents black visually, and meanwhile, the radiation temperature of the engine (~674 K) can be reduced to ~353 K, indicating its application potential in the military field.

## DISCUSSION

In summary, our elaborately designed NPAHP-based skin-like metamaterial enables a highly selective spectrum (high absorptivity of ~0.947 in the visible band and low emissivity of ~0.074/0.045 in the MWIR/LWIR bands) for effective dual-band camouflage at night or in outer space. The ultrathin thickness and periodic penetrative nanopores endow the NPAHP-based metamaterial with skin-like attachability and permeability, desirable for various wearable applications. Meanwhile, benefiting from the extremely low infrared emissivity of NPAHP-based metamaterial, it exhibits excellent high-temperature camouflage performance, capable of reducing the radiation temperature of an object from 678 to 353 K. With flexible multiband modulation, high fabrication throughput, great attachability, permeability, and excellent high-temperature camouflage performance, this skin-like metamaterial is expected to pave a new way in practical applications, such as multiscene/functional camouflage and thermal regulation. Furthermore, it is also expected that in the future, this monolayer NPAHP-based film can be effectively coupled with other materials to enable more-band compatible camouflage and heat dissipation in 5 to 8 μm, thus extending its applicability to broader application scenarios.

## MATERIALS AND METHODS

### Fabrication of the NPAHP-based camouflage film

The NPAHP-based hierarchical structure is fabricated by a simple two-step template method (fig. S1). First, depositing: transferring an AAO template to the e-beam evaporation chamber (TLMDS500) for Au deposition (Au/AAO) (5.0 Å/s for 500 s). It is expected that part of the evaporated Au clusters penetrate into the nanopores, collide, aggregate, and eventually adhere on the sidewalls with random distributions (sizes and shapes), while another part of the Au NPs is attached to the surface, forming a connected Au film (because of the particles more likely to gather in the entrance of the nanopores during the deposition process, the sizes of particles are larger at this location and decrease gradually along the deposition path). Second, etching: put the Au/AAO into the alkaline solution (3 M NaOH aqueous for 5 hours) to completely dissolve the AAO template meanwhile retaining the NPAHP-based structure. Furthermore, to ensure the high-temperature stability of the NPAHP-based camouflage film, a 10-nm-thick SnO_2_ is conformally coated as the protective layer by the ALD system (Savannah S200).

### Radiation temperature measurement

The radiation temperature of the NPAHP-based camouflage film is measured by an infrared camera (FLUKE TiX580) with a detection wavelength range of 8 to 14 μm.

### Optical characterization

The reflection spectra in the ultraviolet–visible–near infrared (UV-Vis-NIR) band (0.28 to 2.5 μm) are measured by a Shimadzu UV-3600 (UV-Vis-NIR) spectrophotometer attached with an integrating sphere (ISR-3100). The reflection spectra in the infrared band (2.5 to 18 μm) are measured by a Thermo Fisher Scientific Nicolet iS50R spectroscope attached with an integrating sphere (4P-GPS-020-SL, Pike).

### SEM characterization

The micromorphology (top view and cross-sectional view) of the NPAHP-based hierarchical structure is characterized by the field emission scanning electron microscope (TESCAN MIRA3, Dual-beam FIB 235, FEI Strata).

### Water vapor transmission test

First, prepare four open glass bottles with a height of 85 mm and a diameter of 14 mm and fill them with 1 g of deionized water and then attach Cu foil, MXene, and NPAHP-based films onto three of the bottles. To quantify their permeability, we tested their mass variation continuously for 7 days at a temperature of 20° to 21°C and a humidity of 25 to 30%.

## Supplementary Materials

### This PDF file includes:

Notes S1 to S16 Figs. S1 to S19 Table S1 References
