# Supplementary material for: Self-assembled skin-like metamaterials for dual-band camouflage
Source: Sci Adv. 2024 Jun 19;10(25):eadl1896. doi: 10.1126/sciadv.adl1896 (PMC11186495; doi:10.1126/sciadv.adl1896)
Supplement: Supplementary file 1 — Notes S1 to S16 Figs. S1 to S19 Table S1 References [file sciadv.adl1896_sm.pdf]

Science Advances
AAAS

Supplementary Materials for

Self-assembled skin-like metamaterials for dual-band camouflage

Shiqi Fang *et al.*

Corresponding author: Jia Zhu, jiazhu@nju.edu.cn; Ning Xu, nxu@nju.edu.cn

Sci. Adv. **10**, eadl1896 (2024)
DOI: 10.1126/sciadv.adl1896

This PDF file includes:

Notes S1 to S16
Figs. S1 to S19
Table S1
References

Supplementary Text

Note S1 Optical simulations

All optical simulations in this manuscript are carried out by the FDTD method. For the NPAHP-based hierarchical structure, three typical structural features are involved, e.g., particles (tens of nanometers), pillars (hundreds of nanometers), and the filling ratio of Au (macroscopic), which respond to different optical effects, respectively. Thus, three simplified optical models are adopted to explore the absorption performance in different bands and then determine the optimal structural parameters.

In Fig. 2A, the LSPR effect of Au NPs dominates the visible absorption. For a single particle, with the increase in aspect ratio, the resonance absorption peak of the particle will red-shift, as shown in Fig. S1. To further study the dependence of absorption performance on particle size distribution (from single-sized particles to multi-sized particles) in the visible band, a 3D multi-particle model is adopted. Here, the particles are considered to be distributed on a pillar surface of H 400 nm, D 150 nm, P 180 nm (Fig. S2). The total number of particles is $N = 60$, and the particles are almost separated from each other. Assuming that the types of particle size are n, thus the detailed particle distribution is:

H_1: $0 \sim 400/n$; $d_2 = 20 \times 1$ nm; (the long axis length (d_2) of the particles is variate, whereas the short axis length (d_1) is fixed at 20 nm) $N_1 = a$;

H_2: $400/n \sim 400/n * 2$; $d_2 = 20 \times 2$ nm; $N_2 = a/2$;

...

H_n: $400/n * (n-1) \sim 400$; $d_2 = 20 \times n$ nm; $N_2 = a/n$;

Here, $N_1 + N_2 + \cdots + N_n = N$, n takes the values 1, 2, 3, 4, 5.

These curves indicate that the visible absorption is pronounced with the broadening of the particle size distribution.

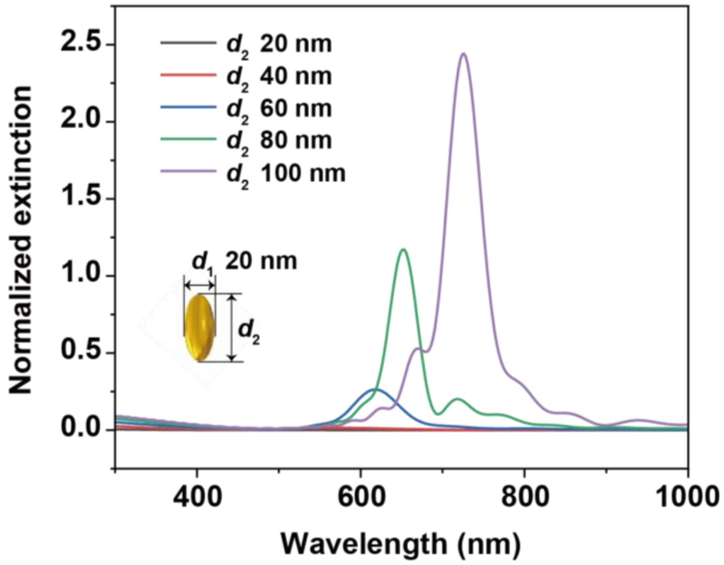

Fig. S1. Calculated normalized extinction cross-section of Au NP with different aspect ratios.

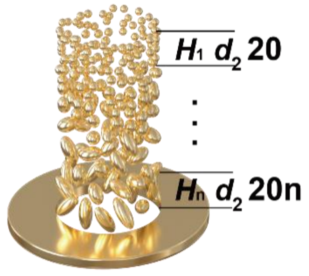

Fig. S2. Particle distribution model in Fig. 2A.

In addition, to demonstrate that the three-dimensional pillar structures formed by the longitudinally distributed Au NPs in the NPAHP structure enable effective light coupling and enhance visible absorptivity by light scattering, we simulate two particle distribution state, i.e., three-dimensional longitudinal distribution and the two-dimensional in-plane distribution. As shown in Fig. S3, the visible absorptivity is further pronounced at the state of three-dimensional distribution. Specifically, for the three-dimensional distribution state, the simulated conditions and the number and size distribution of particles are the same as in the case of n=5 (n refers to the types of particle size) mentioned above. (The only difference is that there are no through-holes in the Au film to avoid the impact of light absorption within confined holes). For the two-dimensional state, the number and size distribution of particles are the same as in

the three-dimensional case, except that the particles are randomly distributed on the plane of the Au film within the same simulated area.

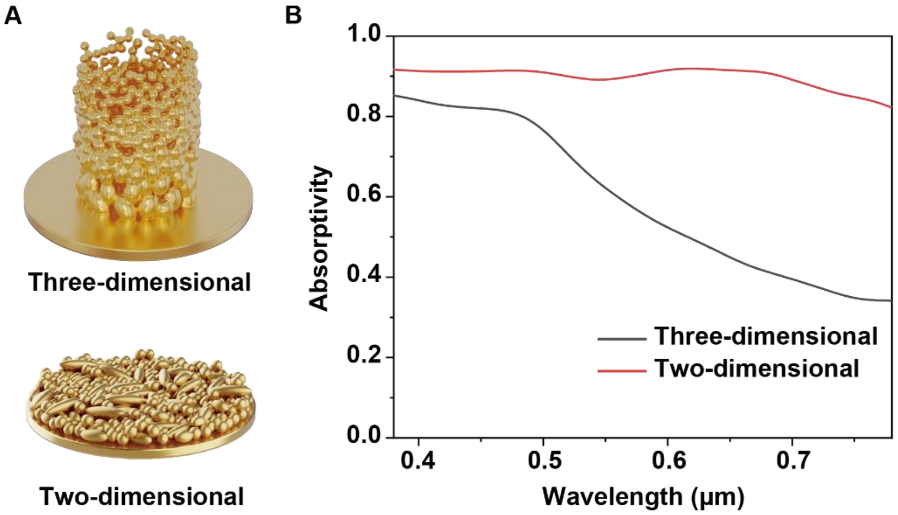

Fig. S3. Absorption performance of particles with different distribution states in the visible band. (**A**) Schematic of particles with different distribution states. (**B**) Absorption spectra of particles with different distribution states.

In Fig. 2B, for the MWIR band, small-sized Au NPs are negligible, thus the optical model is simplified to a simple 3D hollow pillar with a thin and uniform sidewall. To evaluate the dependence of emissivity on the geometry of the pillar (height: H; diameter: D), the detailed parameters in Fig. 2B are D (50 nm; 100 nm; 200 nm; 300 nm; 400 nm;), H (10 nm; 100 nm; 400 nm; 700 nm; 1000 nm), P (250 nm; 300 nm; 400 nm; 500 nm; 600 nm). In addition, another two sets of simulations were performed, i.e., fixed D (400 nm) and P (600 nm), varied H (10 nm; 100 nm; 400 nm; 700 nm; 1000 nm) and fixed H (1000 nm) varied D (50 nm; 100 nm; 200 nm; 300 nm; 400 nm) and corresponding P (250 nm; 300 nm; 400 nm; 500 nm; 600 nm) in Fig. S4. It is observed that having a relatively small feature geometry (D, H) is important.

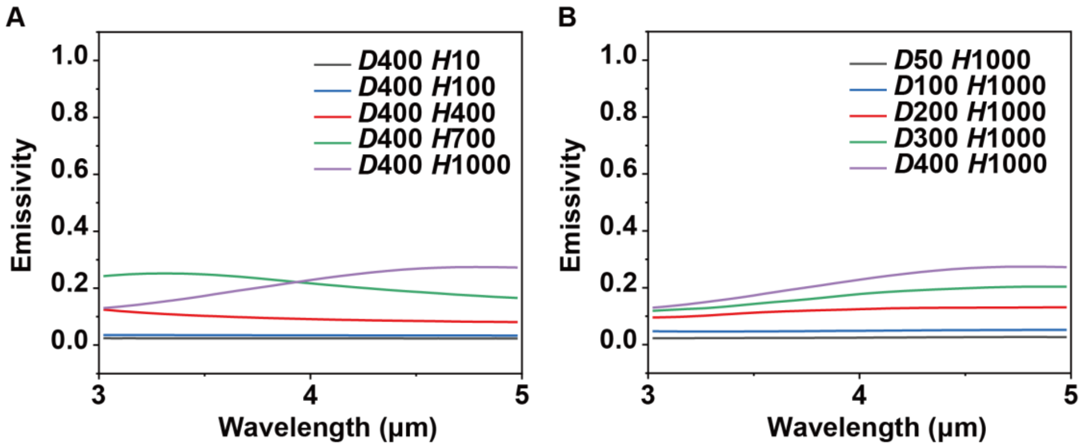

Fig. S4. Optical simulations of NPAHP structure. Dependence of the absorption on the H (fixed D) (**A**) and the D (fixed H) (**B**).

In Fig. 2C, unlike the visible and MWIR bands, for the LWIR band, the enhanced absorption attributed to the characteristic structure can be neglected because the sizes of the particles and pillars are smaller than the subwavelength. Thus, this structure is regarded as a homogeneous medium (A), and the optical properties are dependent on the filling ratio of Au (f). Here, the optical model is further simplified to a homogeneous block to figure out the effect of f on absorption performance. The refractive index n_{a} and extinction coefficient k_{a} of A can be expressed as follows:

$$n_{\mathrm{a}} = n_1 \times f + n_2 \times (1 - f) \tag{S1}$$

$$k_{\mathrm{a}} = k_1 \times f + k_2 \times (1 - f) \tag{S2}$$

$$f = \frac{(D^2 - D_{\mathrm{t}}{}^2) \times H \times f_1 + (P^2 - D^2) \times (H + h)}{P^2 \times (H + h)} \tag{S3}$$

$$D_t = D - 2 \times t \tag{S4}$$

Here, n_1, n_2 are the refractive index of Au and air, respectively; k_1, k_2 are the extinction coefficient of Au and air, respectively; P (600 nm) is the period of the feature physical unit cell; D (400 nm) and D_t (360 nm) are the outside and inside diameters of the pillar, respectively. H and h (250 nm) are the height of the pillar and Au film, respectively; t (20 nm) is the sidewall thickness of the pillar; f_1 (0.5) is defined as the filling ratio of Au at the sidewall. As shown in Fig. 2C, an appropriate Au ratio (＞20%) is necessary to maintain low emission in the LWIR band.

In Fig. 2D, the full-wave electromagnetic simulations are carried out over 0.3-18 μm based on three reasonable and extreme structure models. These models are considered as randomly distributed Au NPs assembled pillars vertically aligned on periodic porous Au film with a thickness of $h = 250$ nm. The detailed modeling parameters for each feature physical unit cell are as follows.

In case 1, the $D = 50$ nm, $H = 10$ nm, $P = 75$ nm, the particle size is the same, $d_2 = 20$ nm ($d_1 = 20$ nm), $N = 20$;

In case 2, the $D = 200$ nm, $H = 400$ nm, $P = 300$ nm, the particle sizes including $d_2 = 20, 40, 60$ nm ($d_1 = 20$ nm), $N = 600$;

In case 3, the $D = 400$ nm, $H = 1000$ nm, $P = 600$ nm, the particle sizes including $d_2 = 20, 40, 60, 80, 100$ nm ($d_1 = 20$ nm), $N = 2100$;

For the three cases, the distribution trend is that the large particles are located at the orifice, and along the direction away from the orifice, the particle sizes gradually decrease.

In the above simulations, the reflection (R) and transmission efficiency (T) are detected by two power monitors, and then the absorption efficiency (A) is calculated by $A = 1 - R - T$. The refractive index n_1 and extinction efficient k_1 of Au used in the simulation are obtained from Ciesie's data.

Note S2 Fabrication of the NPAHP-based camouflage film

The NPAHP-based hierarchical structure is fabricated by a simple two-step template method, as shown in Fig. S5.

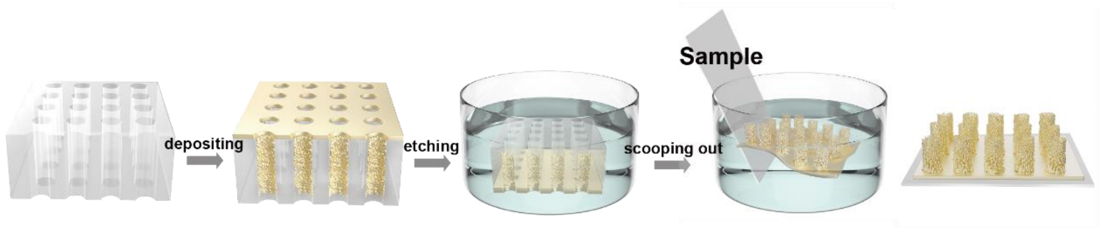

Fig. S5. Fabrication schematic of NPAHP-based camouflage film.

Note S3 SEM characterization of Au/AAO ($D = 50, 120, 390$ nm)

The Au/AAO samples are obtained, after the deposition of Au clusters in AAO templates with three different pore sizes ($D = 50, 120, 390$ nm) (marked as Au/AAO-

50/120/390), and thus the distribution of particles within the nanopores is characterized in Fig. S6.

For Au/AAO-50, the nanopores of the AAO template are prone to be blocked during the process of Au deposition (5.0 Å/s), and only a few dispersed and small particles are distributed near the orifices, as displayed in Fig. S6A and 6D.

For Au/AAO-390, owing to the large pore size, large amounts of evaporated Au clusters penetrate the pores, colliding, aggregating, and finally forming closely-packed NPs, showing a wide range of particle sizes and a deeper penetration depth (Fig. S6B and 6E).

For Au/AAO-120, particle sizes and distribution are the intermediate states between the above two (Fig. S6C and 6F).

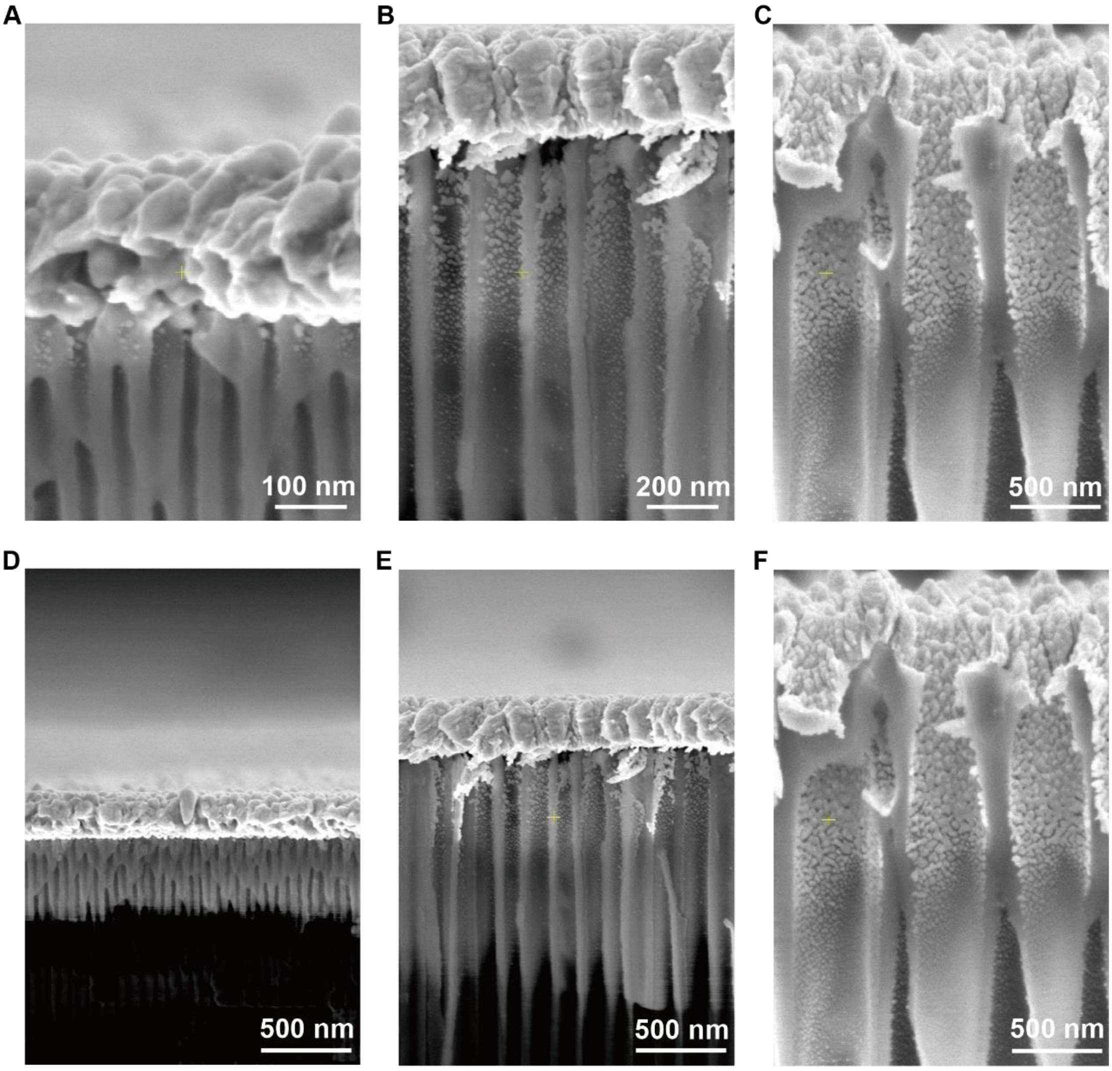

Fig. S6. Cross-section images of Au/AAO. (A-C) SEM images of Au/AAO-50 (A), Au/AAO-120 (B), and Au/AAO-390 (C) at different magnifications. **(D-F)** SEM

images of Au/AAO-50 (D), Au/AAO-120 (E), and Au/AAO-390 (F) at the same magnification.

Note S4 Absorption spectra of Au/AAO-50/120/390

The absorption spectra of deposited Au/AAO samples with different pore sizes are characterized in Fig. S7 (light is incident from the AAO side). The three samples all present high absorption in the infrared band due to the AAO template, lacking the ability of infrared camouflage.

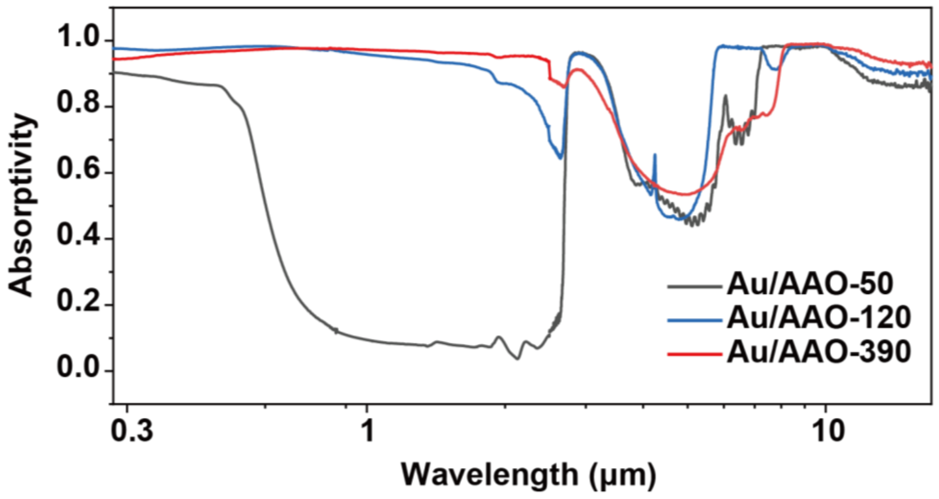

Fig. S7. Absorption spectra of Au/AAO-50/120/390 over 0.28-17 μm.

Note S5 Demonstration of the complete etching of the AAO template

To verify that the AAO template is completely removed during the etching process, XPS analysis was performed (Thermo Scientific, K-Alpha). The XPS spectrum of the etched sample is shown in Fig. S8. Obviously, no characteristic peak of Al 2p appears, indicating that the AAO template is completely dissolved after etching.

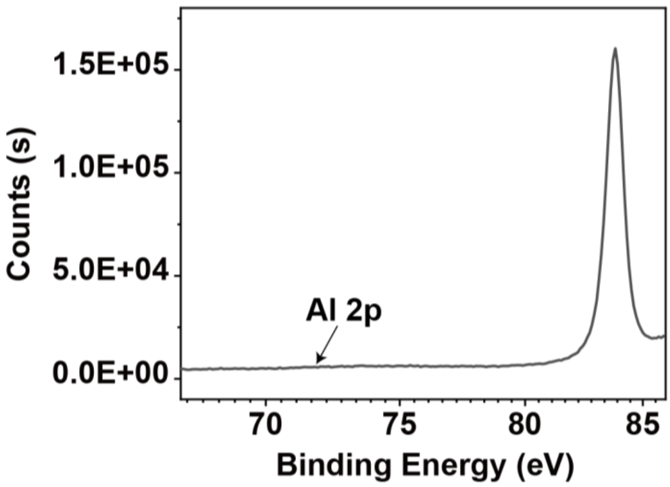

Fig. S8. XPS spectrum of the etched sample.

Note S6 Absorption spectra of AAO, Au/AAO, and NPAHP-based film with a pore size of 120 nm

To illustrate the necessity of etching, the absorption spectra of AAO, Au/AAO, and NPAHP-based film with a pore size of 120 nm are characterized in Fig. S9. For the pure AAO template, it has a low absorption in the visible band, while has a strong absorption due to the lattice vibrations (Al-O) in the infrared band. Once the Au NPs are deposited, this sample shows different optical properties on the deposited side and the backside. The deposited side (Au/AAO-Au) presents the typical spectrum of gold due to the formation of a connected Au film, which visually appears to be golden. For the backside (light is incident from the AAO side, Au/AAO-AAO), in the visible band, the incident light can act on the Au NPs inside the nanopores due to the high visible transmission of the AAO template, thus inducing the LSPR hybridization effect, thereby achieving a high absorption. However, it also presents a high absorption due to the intrinsic lattice vibrations of the AAO template in the infrared band. Therefore, to achieve the desired selective spectrum, it is necessary to remove the AAO template, while retaining the NPAHP-based hierarchical structure.

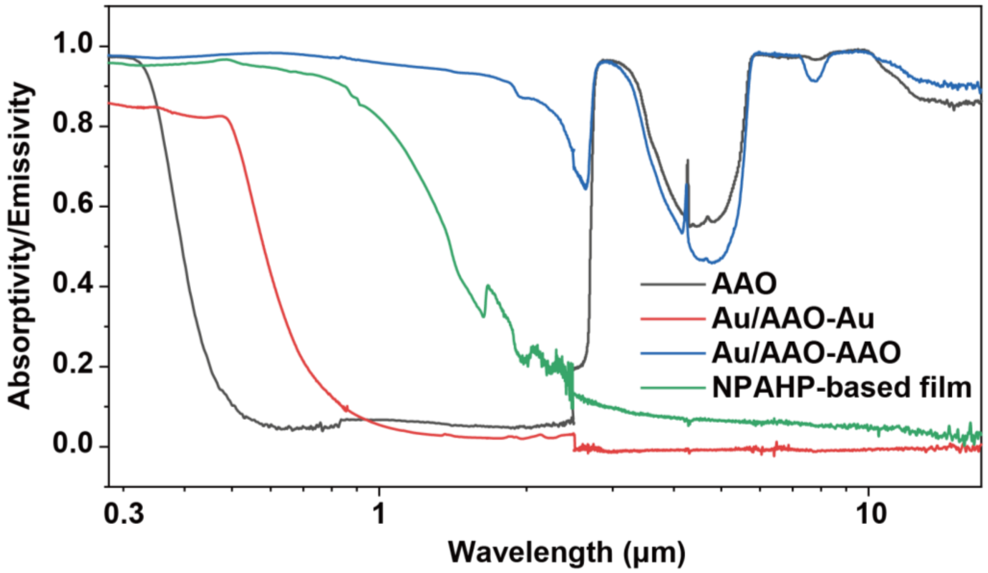

Fig. S9. Absorption spectra of AAO, Au/AAO, and NPAHP-based film with the pore size of 120 nm over 0.28-17 μm.

Note S7 Multiple experiments for NPAHP-50/120/390

To demonstrate the controllability of the NPAHP structures fabricated based on the AAO template method, we conducted multiple experiments and characterized the morphology and optical spectra of NPAHP-120-(1-5). As shown in Fig. S10, the structures of NPAHP-120-(1-5) exhibit excellent uniformity and repeatability. Based on the consistent structures, Fig. S11C and S11D illustrate the spectra consistency of NPAHP-120-(1-5). The average absorptivity in the visible band is 0.943, The average emissivity in the MWIR/LWIR bands is 0.091/0.044.

In addition to NPAHP-120, NPAHP-50/390 was also repeatedly prepared. Their spectra and error analysis are shown in Fig. S11. For the NPAHP-50-(1-5), the average absorptivity in the visible band is 0.293, and the average emissivity in the MWIR/LWIR bands is 0.017/0.011. For the NPAHP-390-(1-5), the average absorptivity in the visible band is 0.973, and the average emissivity in the MWIR/LWIR bands is 0.777/0.245. These spectra all exhibit highly consistent spectra.

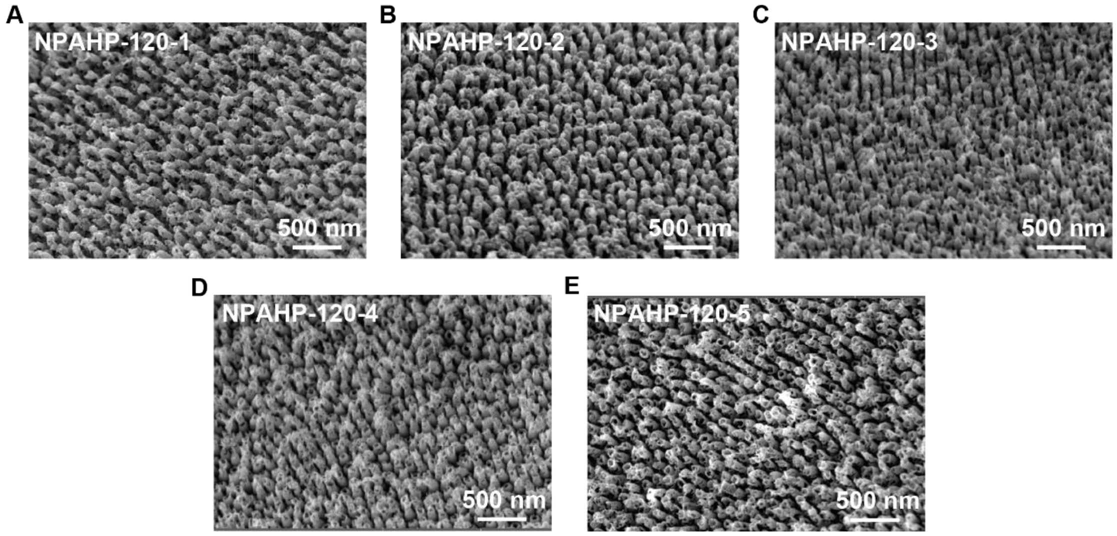

Fig. S10. SEM images for NPAHP-120-(1-5). (**A**) NPAHP-120-1; (**B**) NPAHP-120-2; (**C**) NPAHP-120-3; (**D**) NPAHP-120-4; (**E**) NPAHP-120-5.

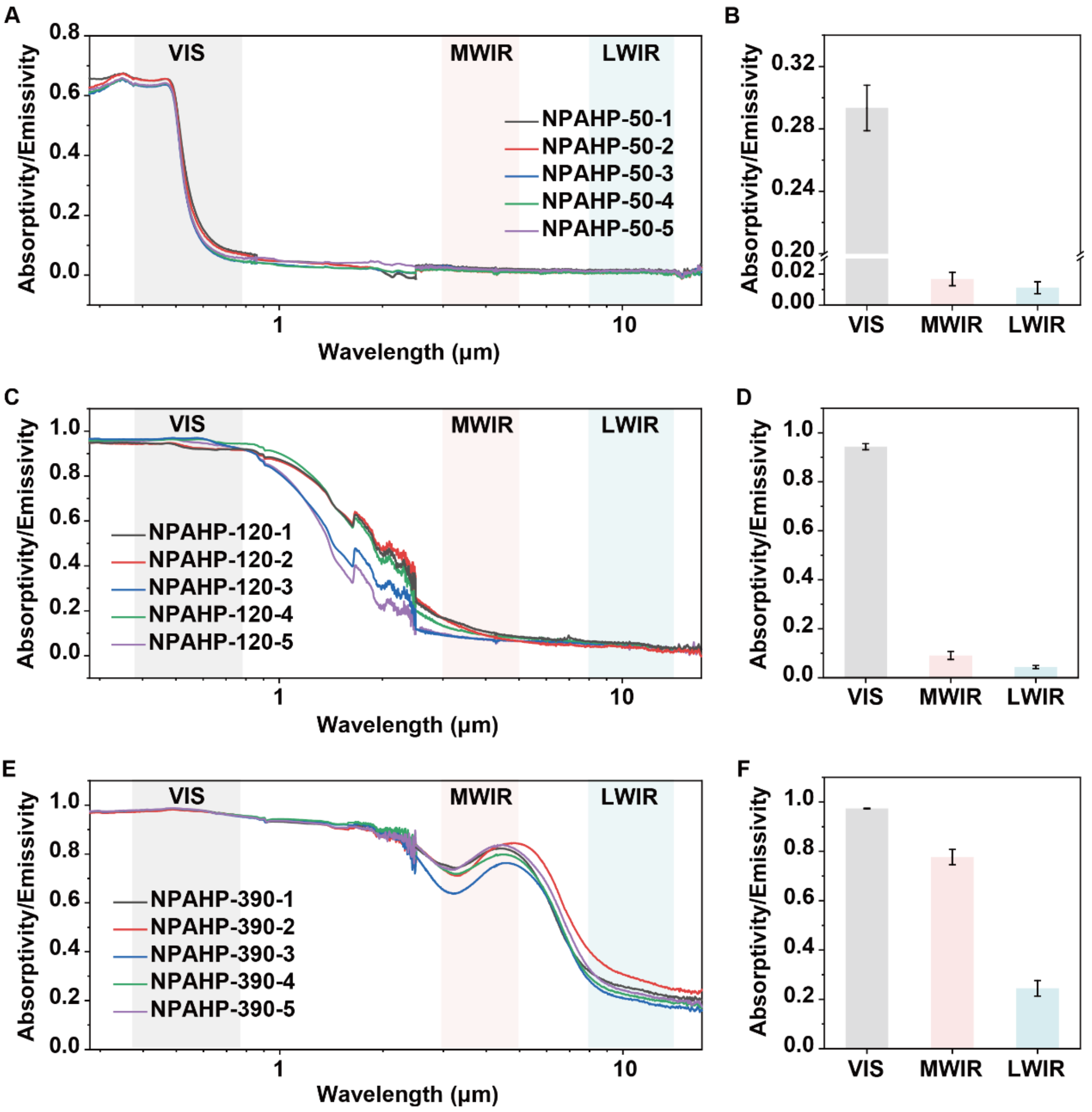

Fig. S11. Absorption spectra and error analysis of absorptivity/emissivity for NPAHP-50/120/390. (**A** and **B**) NPAHP-50; (**C** and **D**) NPAHP-120; (**E** and **F**) NPAHP-390.

Note S8 Absorptivity/Emissivity of NPAHP-120 at different incident angles

Here, we characterized the absorptivity and emissivity of the NPAHP-120 at different incident angles in both visible and infrared bands, as shown in Fig. S12. The results indicate that our NPAHP-120 structure exhibits angle insensitivity over a wide range of angles (0-50°). This is also verified by the infrared photographs at different angles.

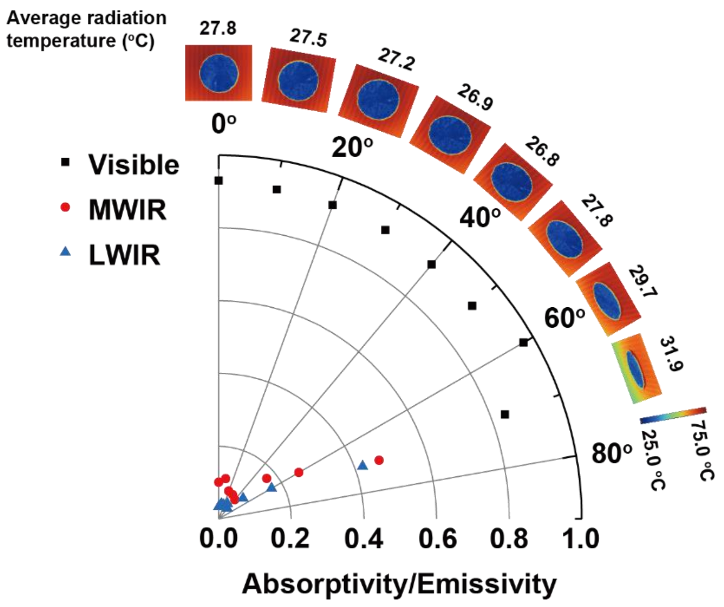

Fig. S12. Absorptivity/Emissivity of the NPAHP-120 in the visible, MWIR, and LWIR bands at different incident angles. The pictures on the top show the infrared photographs of NPAHP-120 taken at different incident angles.

Note S9 Simulated absorption spectra based on experimental structures of NPAHP-50/120/390

Compared with the experimental spectra of NPAHP-50/120/390, it is obviously observed that the absorption features are produced well by the FDTD simulations (Fig. S13). In the simulations, the general outline of the model is similar to that depicted in Note S1, and the specific model parameters such as D, H, P, h, and the distribution of Au NPs are set based on the SEM images in Fig. 2D to 2I.

Specifically, for the the NPAHP-50, the simulated structure is set as an Au film with periodic slight protrusions based on the SEM images, so that semi-ellipsoidal Au particles with $D = 50\,nm$, $H = 10\,nm$, $P = 100\,nm$ are distributed on it, and the thickness of the Au film underneath is $h = 250\,nm$.

For the NPAHP-120, in the simulated structure, the diameter (D), height (H), and periodic (P) of the pillar are set as 120 nm, 300 nm, and 180 nm, and the thickness (h) of the Au film is 250 nm. The Au NPs are set as ellipsoidal, with the short axis length (d_1) fixed at 20 nm and the long axis length (d_2) ranging from 8 nm to 72 nm. The total number (N) of particles is 600. The distribution trend is that the large particles

are located at the orifice, and along the direction away from the orifice, the particle sizes gradually decrease.

For the NPAHP-390, in the simulated structure, the thickness (h) of the Au film, the short axis length (d_1) and the distribution trend of the particles are all consistent with those of the NPAHP-120. The diameter (D), height (H), and periodic (P) of the pillar are set as 390 nm, 1000 nm, and 550 nm. The long axis length (d_2) of particles range from 10 nm to 150 nm, and the total number (N) of particles is 2100.

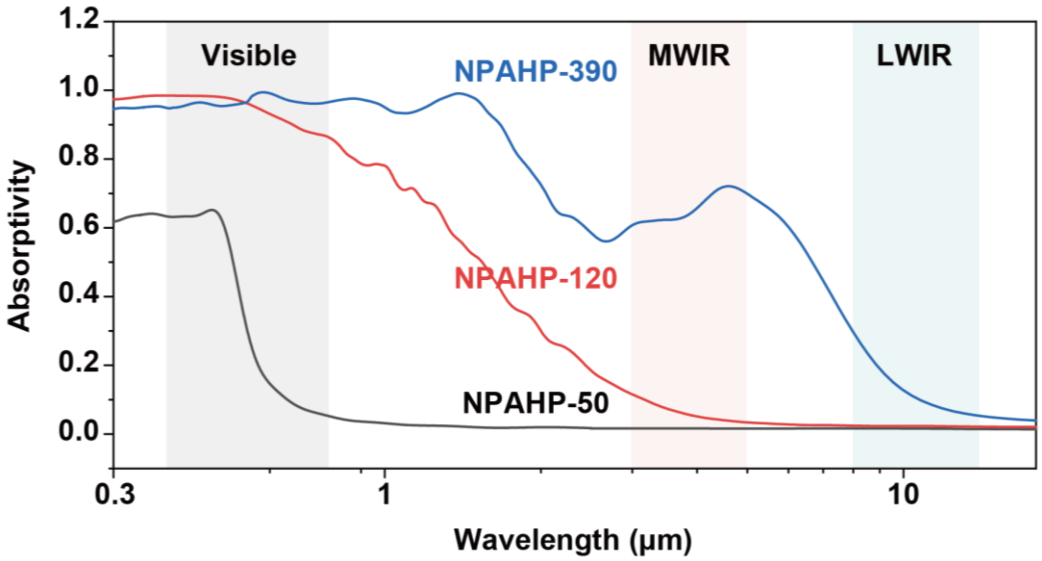

Fig. S13. Simulated absorption spectra based on experimental structures of NPAHP-50/120/390.

Note S10 Characterization of adhesiveness of NPAHP-120 film

The NPAHP-120 film exhibits good adhesiveness due to the close contact and the Van der Waals between the sample and the substrate. As shown in Fig. S14, when the NPAHP-120 film is attached to a glass substrate, the sample will also not be detached from the substrate at different tilt angles or even when completely inversed. Furthermore, for simulating the tougher condition, the wind speed approaching 3.0 m/s is introduced, and it is observed that the sample still does not detach from the substrate.

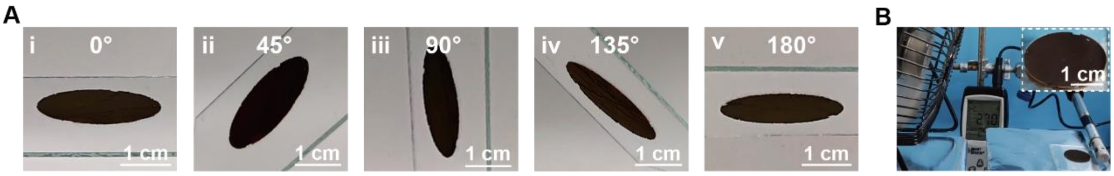

Fig. S14. Optical photographs of the NPAHP-120 film in different states. (**A**) At different tilt angles (0°-180°). (**B**) Under wind speeds approaching 3.0 m/s (inset shows an enlarged view of the NPAHP film under wind blowing).

Note S11 Characterization of air permeability of NPAHP-120 film

To further quantitatively assess the permeability of NPAHP-120 film, an air permeability test is also conducted. As shown in Fig. S15, our NPAHP-120 film exhibits similar good air permeability as commercial cotton.

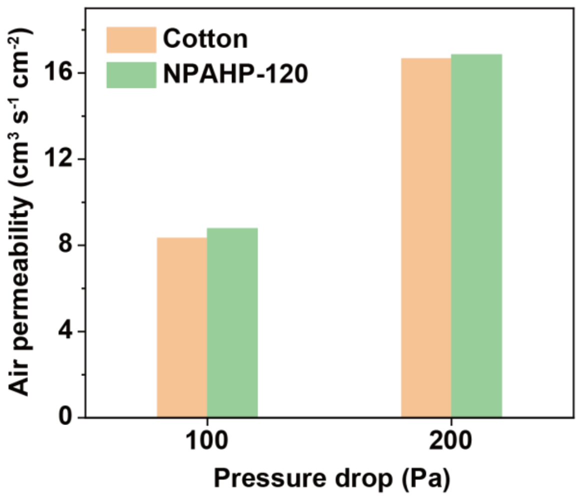

Fig. S15. Air permeability of NPAHP-120 film and commercial cotton. (The data for commercial cotton (~300 μm) is referred from the ref. (*64)*)

Note S12 Characterization of the durability of NPAHP-120 film

We also evaluate the durability of NPAHP-120 film in two aspects. Firstly, the spectra of NPAHP-120 film before and after being placed in different environmental temperatures and humidity over one month are characterized (Fig. S16), which show that its optical properties remain almost unchanged (slight changes arise from the testing errors), proving its good spectral stability and durability. Besides, to further demonstrate its durability for the scenarios involving repetitive bending of joints, the NPAHP-120 film is folded 100 times to simulate the repeated usage process, as shown in Fig. S17. The characterizations show that the optical photographs and spectra of the NPAHP-120 film remain unchanged before and after folding.

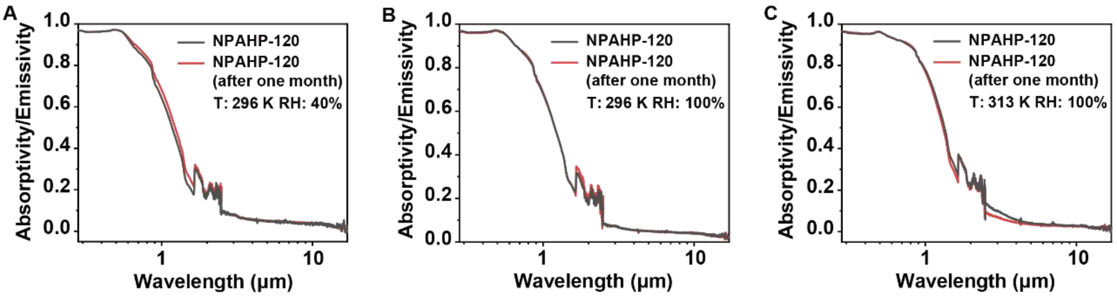

Fig. S16. Absorption spectra of the NPAHP-120 film before and after one month under three typical environmental temperature and humidity conditions. (**A**) T: 296 K, RH: 40%; (**B**) T: 296 K, RH: 100%; (**C**) T: 313 K, RH: 100%.

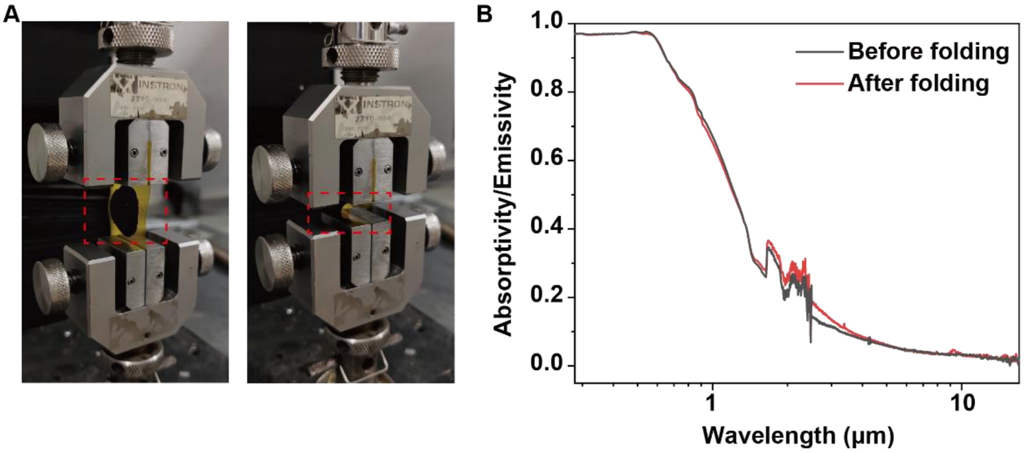

Fig. S17. Performance of NPAHP-120 film after folding it over 100 times. (**A**) Optical photographs of the NPAHP-120 film during the folding test. (**B**) Absorption spectra of the NPAHP-120 film before and after folding.

Note S13 Calculations for the energy contribution to the detected power by the infrared camera

In Fig. 5A, the energy contribution of radiative power from the target (P_{rad}) and the reflective power of ambient radiation by the target (P_{ref}) to the detected power (P_{det}) and the dependence of P_{rad} and P_{ref} on real temperature (T_1) and emissivity (ε_1) of the target are depicted.

The detailed calculation process is as follows:

$$P_{\mathrm{det}} = P_{\mathrm{rad}}(\varepsilon_1, T_1) + P_{\mathrm{ref}}(\varepsilon_1, \varepsilon_{\mathrm{a}}, T_{\mathrm{a}}) \quad \text{(S5)}$$

$$P_{\mathrm{rad}}(\varepsilon_1, T_1) = \int_8^{14} \varepsilon_1 \; I_{\mathrm{BB}}\,(T_1, \lambda)\; d\lambda \quad \text{(S6)}$$

$$P_{\mathrm{ref}}(\varepsilon_1, \varepsilon_{\mathrm{a}}, T_{\mathrm{a}}) = \int_8^{14} (1 - \varepsilon_1)\; \varepsilon_{\mathrm{a}}\; I_{\mathrm{BB}}\,(T_{\mathrm{a}}, \lambda)\; d\lambda \quad \text{(S7)}$$

where, T_a and ε_a are the ambient temperature and emissivity, respectively. The range of 8-14μm is the detection wavelength of the IR camera. For most natural environments, the ambient emissivity is close to a blackbody, thus ε_a and T_a are supposed to be 1.0 and 298 K.

I_{BB} can be depicted by Planck's law:

$$I_{BB}\,(T,\lambda) = \frac{8\pi hc}{\lambda^5} \times \frac{1}{e^{\frac{hc}{\lambda kT}}-1} \tag{S8}$$

where h is the Planck constant, k is the Boltzmann constant, and c is the speed of light in the air.

Note S14 Calculations for the dependence of the radiation temperature of the target on real temperature and emissivity

The default emissivity ε_{IR} of the detected target is usually 0.9 in the IR camera. The program in the IR camera can convert the detected power P_{det} into the radiation temperature T_r of the target. The specific relationship is as follows:

$$P_{det} = P_{det}(\varepsilon_{IR}, T_{rad}) = \int_8^{14} \varepsilon_{IR}\, I_{BB}\,(T_r, \lambda)\, d\lambda \tag{S9}$$

Here, for a definite scenario, the values of P_{det} and ε_{IR} are determined, and then T_r can be derived.

Note S15 Absorption spectra of NPAHP-120 in different states

Here, we characterized three spectra corresponding to different states of the NPAHP-120 film. They are the pristine NPAHP-120, the NPAHP-120 coated with SnO_2 but not annealed, and the NPAHP-120 coated with SnO_2 and then annealed for 1 hour at 673 K in air, respectively.

As shown in Fig. S18, the optical properties of the samples in the three states remain almost unchanged. The negligible impact on optical performance is ascribed to the reason that the SnO_2 layer coated by the ALD method is ultra-thin (10 nm) and dense, which hardly affects the spectra of NPAHP-120 in the visible and infrared bands. Moreover, the SnO_2 coating layer protects the structure of the Au NPs under high temperatures, making the optical property of the NPAHP-120 film well maintain after annealing.

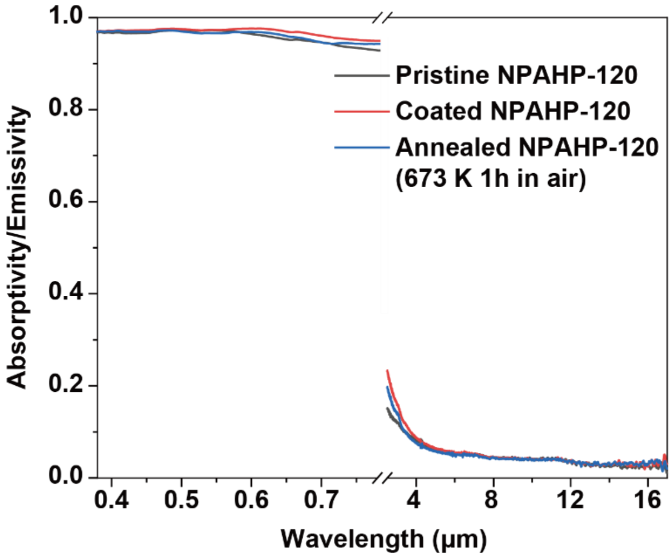

Fig. S18. Absorption spectra of the pristine NPAHP-120, the coated NPAHP-120, and the annealed NPAHP-120 over 0.28-17 μm.

Note S16 SEM characterization of the NPAHP-120 coated with a protective layer before and after annealing (673 K; 1 h; air)

The micro-morphology of the NPHAP-120-based hierarchical structure before and after annealing is characterized, as shown in Fig. S19, which demonstrates that the protective layer (10 nm SnO_2) is capable of maintaining structural stability at 673 K for 1 h.

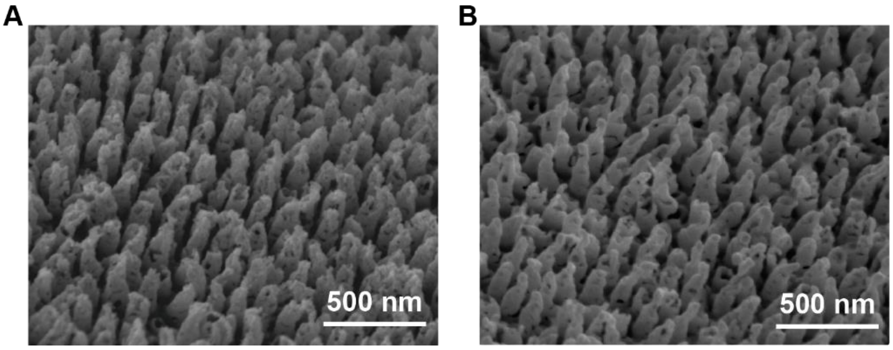

Fig. S19. SEM images of the NPAHP-120. (**A**) Coated NPAHP-120; (**B**) Annealed NPAHP-120.

Table S1. Summary of important parameters of recently reported multi-spectral camouflage materials.

Materials	Structure design	Fabrication complexity	Absorptivity/Color in the visible band	Emissivity in the MWIR band	Emissivity in the LWIR band	Laser	Microwave	High-temperature camouflage performance (the actual temperature of camouflage material T_1-corresponding radiation temperature T_r)	Ref.
ZnS/Ge multilayer with Cu-ITO-Cu metasurface	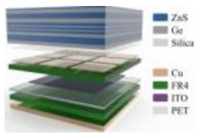	17 layers	Green; Blue; Purple	0.11	0.12	~0.8 (1.55 µm; 10.6 µm)	0.9 (8-12 GHz)	——	*(38)*
Au-ITO-Si_3N_4-SiO_2	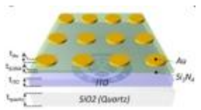	4 layers	Transparent	0.36	0.25	——	——	——	*(54)*
Si-Al	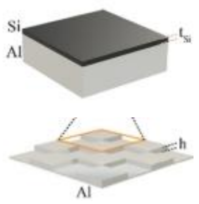	2 layers	Gray; Red; Yellow	——	0.17	——	0.9 (8-13 GHz)	——	*(41)*

Silicon-based fractal nanostructures	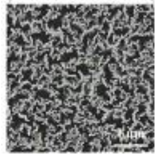	1 layer	0.97	——	0.33	——	——	——	*(34)*
Au-PET-resistive film-foam-metal	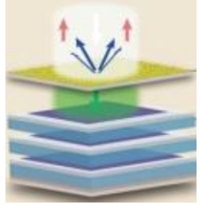	9 layers	——	——	0.2	~0.8 (0.9-1.2 μm)	0.9 (2.7-26 GHz)	——	*(55)*
TiO_2-Ge-SiO_2 (Theoretical work)	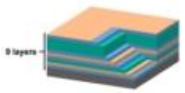	9 layers	Green; Blue; Yellow	0.12	0.21	0.99 (1.06 μm; 10.6μm)	——	——	*(58)*
Ge-TiO_2-SiO_2 (Theoretical work)	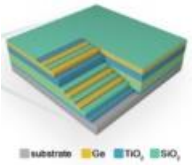	12 layers	——	0.14	0.21	——	——	——	*(57)*
Ge-YbF_3	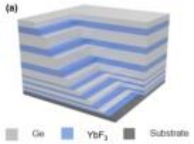	14 layers	0.79	0.06	0.01	——	——	410 K (T_1)-313 K (T_r) (the temperature of the object's surface is 873 K, as silica aerogel is placed between the object and the Ge-YbF_3 multilayer emitter)	*(56)*
Si-GST-Au	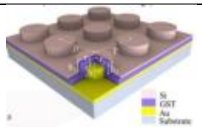	4 layers	Gray; Blue; Red	0.25	0.77	0.9 (10.6 μm)	——	——	*(37)*

Ag-Ge	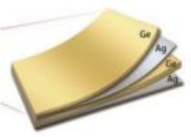	4 layers	——	0.18	0.31	——	——	473 K (T_1)<br>(T_r is not reported)	*(65)*
SiO_2-Ag-ZnS	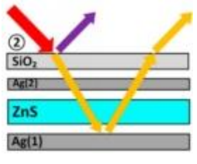	4 layers	Yellow, Navy, Cyan	0.05	0.05	——	——	——	*(66)*
Ge/ZnS	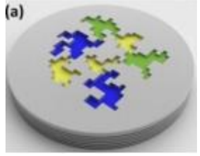	13 layers	Blue; Yellow; Green; Gray	——	0.07	——	——	——	*(35)*
TiAlN-Au-PE	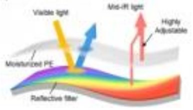	3 layers	Blue; Red; Green	——	0.09	——	——	——	*(67)*
TiO_2-Au-TiO_2	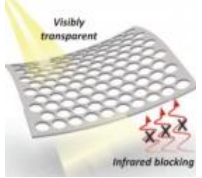	4 layers	Transparent	——	0.2	——	——	523 K (T_1)<br>(T_r is not reported)	*(68)*
ZnO-Ag-ZnO	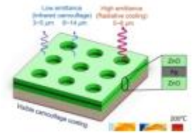	4 layers	Transparent	~0.4	~0.4	——	——	453 K (T_1)-364.3 K (T_r)	*(69)*
MXene	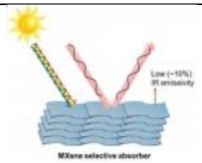	1 layer	~0.9	~0.3	~0.17	——	——	——	*(43)*

MXene	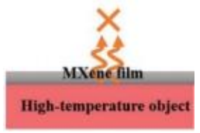	1 layer	——	——	0.19	——	——	783.9 K (T_1)-490 K (T_r)	*(49)*
Ge-ZnS	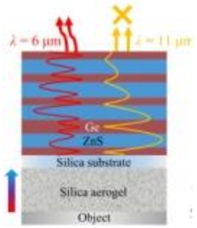	10 layers	——	~0.4	0.078	——	——	409.8 K (T_1)-310.4 K (T_r) (the temperature of the object's surface is 873 K, as silica aerogel is placed between the object and the Ge-ZnS multilayer emitter)	*(48)*
Au	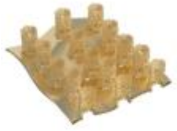	1 layer	~0.947	0.074	0.045	——	——	678 K (T_1)- 353 K (T_r)	Our work

REFERENCES AND NOTES

1 W. Cai, V. M. Shalaev, *Optical Metamaterials: Fundamentals and Applications* (Springer, 2010).

2 V. M. Shalaev, Optical negative-index metamaterials. *Nat. Photonics* **1**, 41–48 (2007).

3 C. M. Soukoulis, M. Wegener, Past achievements and future challenges in the development of three-dimensional photonic metamaterials. *Nat. Photonics* **5**, 523–530 (2011).

4 S. Xiao, V. P. Drachev, A. V. Kildishev, X. Ni, U. K. Chettiar, H.-K. Yuan, V. M. Shalaev, Loss-free and active optical negative-index metamaterials. *Nature* **466**, 735–738 (2010).

5 X. Ni, Z. J. Wong, M. Mrejen, Y. Wang, X. Zhang, An ultrathin invisibility skin cloak for visible light. *Science* **349**, 1310–1314 (2015).

6 W. Cai, U. K. Chettiar, A. V. Kildishev, V. M. Shalaev, Optical cloaking with metamaterials. *Nat. Photonics* **1**, 224–227 (2007).

7 J. A. Bossard, L. Lin, S. Yun, L. Liu, D. H. Werner, T. S. Mayer, Near-ideal optical metamaterial absorbers with super-octave bandwidth. *ACS Nano* **8**, 1517–1524 (2014).

8 N. Liu, T. Weiss, M. Mesch, L. Langguth, U. Eigenthaler, M. Hirscher, C. Sonnichsen, H. Giessen, Planar metamaterial analogue of electromagnetically induced transparency for plasmonic sensing. *Nano Lett.* **10**, 1103–1107 (2010).

9 L. Liu, X. Zhang, M. Kenney, X. Su, N. Xu, C. Ouyang, Y. Shi, J. Han, W. Zhang, S. Zhang, Broadband metasurfaces with simultaneous control of phase and amplitude. *Adv. Mater.* **26**, 5031–5036 (2014).

10 N. Meinzer, W. L. Barnes, I. R. Hooper, Plasmonic meta-atoms and metasurfaces. *Nat. Photonics* **8**, 889–898 (2014).

11 J. Valentine, S. Zhang, T. Zentgraf, E. Ulin-Avila, D. A. Genov, G. Bartal, X. Zhang, Three-dimensional optical metamaterial with a negative refractive index. *Nature* **455**, 376–379 (2008).

12 T. Søndergaard, S. M. Novikov, T. Holmgaard, R. L. Eriksen, J. Beermann, Z. Han, K. Pedersen, S. I. Bozhevolnyi, Plasmonic black gold by adiabatic nanofocusing and absorption of light in ultra-sharp convex grooves. *Nat. Commun.* **3**, 969 (2012).

13 X. Ni, A. V. Kildishev, V. M. Shalaev, Metasurface holograms for visible light. *Nat. Commun.* **4**, 2807 (2013).

14 Y. Yang, H. Fu, H. Su, S. Chen, S. Wu, J. Liang, T. Wei, Y. Wang, S. Zhu, J. Zhu, L. Zhou, Sodium-based concave metasurfaces for high performing plasmonic optical filters by templated spin-on-sodiophobic-glass. *Adv. Mater.* **35**, e2300272 (2023).

15 N. Liu, H. Guo, L. Fu, S. Kaiser, H. Schweizer, H. Giessen, Three-dimensional photonic metamaterials at optical frequencies. *Nat. Mater.* **7**, 31–37 (2008).

16 N. Liu, L. Langguth, T. Weiss, J. Kästel, M. Fleischhauer, T. Pfau, H. Giessen, Plasmonic analogue of electromagnetically induced transparency at the Drude damping limit. *Nat. Mater.* **8**, 758–762 (2009).

17 N. Liu, H. Liu, S. Zhu, H. Giessen, Stereometamaterials. *Nat. Photonics* **3**, 157–162 (2009).

18 M. Kim, K. Yao, G. Yoon, I. Kim, Y. Liu, J. Rho, A broadband optical diode for linearly polarized light using symmetry-breaking metamaterials. *Adv. Opt. Mater.* **5**, 1700600 (2017).

19 J. Liang, Y. Jin, H. Yu, X. Chen, L. Zhou, P. Huo, Y. Zhang, H. Ma, Y. Jiang, B. Zhu, T. Xu, H. Liu, S. Zhu, J. Zhu, Lithium-plasmon-based low-powered dynamic color display. *Natl. Sci. Rev.* **10**, nwac120 (2023).

20 M. Hentschel, M. Schäferling, X. Duan, H. Giessen, N. Liu, Chiral plasmonics. *Sci. Adv.* **3**, e1602735 (2017).

21 A. Kuzyk, M. J. Urban, A. Idili, F. Ricci, N. Liu, Selective control of reconfigurable chiral plasmonic metamolecules. *Sci. Adv.* **3**, e1602803 (2017).

22 S. Yang, X. Ni, X. Yin, B. Kante, P. Zhang, J. Zhu, Y. Wang, X. Zhang, Feedback-driven self-assembly of symmetry-breaking optical metamaterials in solution. *Nat. Nanotechnol.* **9**, 1002–1006 (2014).

23 J. K. Tong, X. Huang, S. V. Boriskina, J. Loomis, Y. Xu, G. Chen, Infrared-transparent visible-opaque fabrics for wearable personal thermal management. *ACS Photonics* **2**, 769–778 (2015).

24 P.-C. Hsu, A. Y. Song, P. B. Catrysse, C. Liu, Y. Peng, J. Xie, S. Fan, Y. Cui, Radiative human body cooling by nanoporous polyethylene textile. *Science* **353**, 1019–1023 (2016).

25 Z. Ma, D. Zhao, C. She, Y. Yang, R. Yang, Personal thermal management techniques for thermal comfort and building energy saving. *Mater. Today Phys.* **20**, 100465 (2021).

26 B. Zhu, W. Li, Q. Zhang, D. Li, X. Liu, Y. Wang, N. Xu, Z. Wu, J. Li, X. Li, P. B. Catrysse, W. Xu, S. Fan, J. Zhu, Subambient daytime radiative cooling textile based on nanoprocessed silk. *Nat. Nanotechnol.* **16**, 1342–1348 (2021).

27 S. Wang, T. Jiang, Y. Meng, R. Yang, G. Tan, Y. Long, Scalable thermochromic smart windows with passive radiative cooling regulation. *Science* **374**, 1501–1504 (2021).

28 X. Li, C. Cao, C. Liu, W. He, K. Wu, Y. Wang, B. Xu, Z. Tian, E. Song, J. Cui, G. Huang, C. Zheng, Z. Di, X. Cao, Y. Mei, Self-rolling of vanadium dioxide nanomembranes for enhanced multi-level solar modulation. *Nat. Commun.* **13**, 7819 (2022).

29 S.-Z. Sheng, J.-L. Wang, B. Zhao, Z. He, X.-F. Feng, Q.-G. Shang, C. Chen, G. Pei, J. Zhou, J.-W. Liu, S.-H. Yu, Nanowire-based smart windows combining electro-and thermochromics for dynamic regulation of solar radiation. *Nat. Commun.* **14**, 3231 (2023).

30 C. Lin, J. Hur, C. Y. Chao, G. Liu, S. Yao, W. Li, B. Huang, All-weather thermochromic windows for synchronous solar and thermal radiation regulation. *Sci. Adv.* **8**, eabn7359 (2022).

31 Y. Li, J. Hao, H. Song, F. Zhang, X. Bai, X. Meng, H. Zhang, S. Wang, Y. Hu, J. Ye, Selective light absorber-assisted single nickel atom catalysts for ambient sunlight-driven CO_2 methanation. *Nat. Commun.* **10**, 2359 (2019).

32 M. Cai, Z. Wu, Z. Li, L. Wang, W. Sun, A. A. Tountas, C. Li, S. Wang, K. Feng, A.-B. Xu, S. Tang, A. Tavasoli, M. Peng, W. Liu, A. S. Helmy, L. He, G. A. Ozin, X. Zhang, Greenhouse-inspired supra-photothermal CO_2 catalysis. *Nat. Energy* **6**, 807–814 (2021).

33 Y. Li, X. Bai, D. Yuan, F. Zhang, B. Li, X. San, B. Liang, S. Wang, J. Luo, G. Fu, General heterostructure strategy of photothermal materials for scalable solar-heating hydrogen production without the consumption of artificial energy. *Nat. Commun.* **13**, 776 (2022).

34 I. Chang, T. Kim, N. Lee, J. Nam, J.-S. Lim, M. Yun, H. H. Cho, Multispectral optical confusion system: Visible to infrared coloration with fractal nanostructures. *ACS Appl. Mater. Interfaces* **14**, 28337–28347 (2022).

35 D. Qi, F. Chen, X. Wang, H. Luo, Y. Cheng, X. Niu, R. Gong, Effective strategy for visible-infrared compatible camouflage: Surface graphical one-dimensional photonic crystal. *Opt. Lett.* **43**, 5323–5326 (2018).

36 M. Li, D. Liu, H. Cheng, L. Peng, M. Zu, Manipulating metals for adaptive thermal camouflage. *Sci. Adv.* **6**, eaba3494 (2020).

37 M. Pan, Y. Huang, Q. Li, H. Luo, H. Zhu, S. Kaur, M. Qiu, Multi-band middle-infrared-compatible camouflage with thermal management via simple photonic structures. *Nano Energy* **69**, 104449 (2020).

38 H. Zhu, Q. Li, C. Tao, Y. Hong, Z. Xu, W. Shen, S. Kaur, P. Ghosh, M. Qiu, Multispectral camouflage for infrared, visible, lasers and microwave with radiative cooling. *Nat. Commun.* **12**, 1805 (2021).

39 J. Yang, X. Zhang, X. Zhang, L. Wang, W. Feng, Q. Li, Beyond the visible: Bioinspired infrared adaptive materials. *Adv. Mater.* **33**, e2004754 (2021).

40 T. Kim, J.-Y. Bae, N. Lee, H. H. Cho, Hierarchical metamaterials for multispectral camouflage of infrared and microwaves. *Adv. Funct. Mater.* **29**, 1807319 (2019).

41 Y. Huang, Y. Zhu, B. Qin, Y. Zhou, R. Qin, P. Ghosh, M. Qiu, Q. Li, Hierarchical visible-infrared-microwave scattering surfaces for multispectral camouflage. *Nanophotonics* **11**, 3613–3622 (2022).

42 J. Teyssier, S. V. Saenko, D. Van Der Marel, M. C. Milinkovitch, Photonic crystals cause active colour change in chameleons. *Nat. Commun.* **6**, 6368 (2015).

43 Y. Li, C. Xiong, H. Huang, X. Peng, D. Mei, M. Li, G. Liu, M. Wu, T. Zhao, B. Huang, 2D $Ti_3C_2T_x$ MXenes: Visible black but infrared white materials. *Adv. Mater.* **33**, e2103054 (2021).

44 C. Xu, G. T. Stiubianu, A. A. Gorodetsky, Adaptive infrared-reflecting systems inspired by cephalopods. *Science* **359**, 1495–1500 (2018).

45 O. Salihoglu, H. B. Uzlu, O. Yakar, S. Aas, O. Balci, N. Kakenov, S. Balci, S. Olcum, S. Süzer, C. Kocabas, Graphene-based adaptive thermal camouflage. *Nano Lett.* **18**, 4541–4548 (2018).

46 S. Hong, S. Shin, R. Chen, An adaptive and wearable thermal camouflage device. *Adv. Funct. Mater.* **30**, 1909788 (2020).

47 S. P. Mahulikar, G. A. Rao, P. S. Kolhe, Infrared signatures of low-flying aircraft and their rear fuselage skin's emissivity optimization. *J. Aircraft* **43**, 226–232 (2006).

48 H. Zhu, Q. Li, C. Zheng, Y. Hong, Z. Xu, H. Wang, W. Shen, S. Kaur, P. Ghosh, M. Qiu, High-temperature infrared camouflage with efficient thermal management. *Light Sci. Appl.* **9**, 60 (2020).

49 L. Li, M. Shi, X. Liu, X. Jin, Y. Cao, Y. Yang, W. Wang, J. Wang, Ultrathin titanium carbide (MXene) films for high-temperature thermal camouflage. *Adv. Funct. Mater.* **31**, 2101381 (2021).

50 Y. Peng, J. Chen, A. Y. Song, P. B. Catrysse, P.-C. Hsu, L. Cai, B. Liu, Y. Zhu, G. Zhou, D. S. Wu, H. R. Lee, S. Fan, Y. Cui, Nanoporous polyethylene microfibres for large-scale radiative cooling fabric. *Nat. Sustain.* **1**, 105–112 (2018).

51 R. Hu, Y. Liu, S. Shin, S. Huang, X. Ren, W. Shu, J. Cheng, G. Tao, W. Xu, R. Chen, X. Luo, Emerging materials and strategies for personal thermal management. *Adv. Energy Mater.* **10**, 1903921 (2020).

52 C. Chen, Y. Kuang, L. Hu, Challenges and opportunities for solar evaporation. *Joule* **3**, 683–718 (2019).

53 L. Zhu, M. Gao, C. K. N. Peh, G. W. Ho, Recent progress in solar-driven interfacial water evaporation: Advanced designs and applications. *Nano Energy* **57**, 507–518 (2019).

54 N. Lee, J.-S. Lim, I. Chang, D. Lee, H. H. Cho, Transparent metamaterials for multispectral camouflage with thermal management. *Int. J. Heat Mass Transf.* **173**, 121173 (2021).

55 X. Feng, M. Pu, F. Zhang, R. Pan, S. Wang, J. Gong, R. Zhang, Y. Guo, X. Li and X. Ma, Large-area low-cost multiscale-hierarchical metasurfaces for multispectral compatible camouflage of dual-band lasers, infrared and microwave. *Adv. Funct. Mater.* **32**, 2205547 (2022).

56 L. Huang, H. Li, Z. Li, W. Zhang, C. Ma, C. Zhang, Y. Wei, L. Zhou, X. Li, Z. Cheng, Multiband camouflage design with thermal management. *Photonics Res.* **11**, 839–851 (2023).

57 L. Wang, Y. Yang, X. Tang, B. Li, Y. Hu, Y. Zhu, H. Yang, Combined multi-band infrared camouflage and thermal management via a simple multilayer structure design. *Opt. Lett.* **46**, 5224–5227 (2021).

58 L. Wang, S. Zhang, J. Dong, L. Ma, C. Zheng, W. Zhang, L. Liu, Multi-band infrared camouflage compatible with radiative cooling and visible colors via a simple multilayer film structure. *Opt. Mater. Express* **13**, 2746–2758 (2023).

59 H. Wang, H. Alshehri, H. Su, L. Wang, Design, fabrication and optical characterizations of large-area lithography-free ultrathin multilayer selective solar coatings with excellent thermal stability in air. *Sol. Energy Mater. Sol. Cells* **174**, 445–452 (2018).

60 Y. Li, C. Lin, D. Zhou, Y. An, D. Li, C. Chi, H. Huang, S. Yang, C. Y. Tso, C. Y. H. Chao, Scalable all-ceramic nanofilms as highly efficient and thermally stable selective solar absorbers. *Nano Energy* **64**, 103947 (2019).

61 M. Han, D. Zhang, C. E. Shuck, Y. Gogotsi, Ultralow and selective infrared emission from MXenes. arXiv:2105.04011 (2021).

62 E. Prodan, C. Radloff, N. J. Halas, P. Nordlander, A hybridization model for the plasmon response of complex nanostructures. *Science* **302**, 419–422 (2003).

63 J. A. Fan, C. Wu, K. Bao, J. Bao, R. Bardhan, N. J. Halas, V. N. Manoharan, P. Nordlander, G. Shvets, F. Capasso, Self-assembled plasmonic nanoparticle clusters. *Science* **328**, 1135–1138 (2010).

64 X. Wu, J. Li, Q. Jiang, W. Zhang, B. Wang, R. Li, S. Zhao, F. Wang, Y. Huang, P. Lyu, Y. Zhao, J. Zhu, R. Zhang, An all-weather radiative human body cooling textile. *Nat. Sustain.* **6**, 1446–1454 (2023).

65 L. Peng, D. Liu, H. Cheng, S. Zhou, M. Zu, A multilayer film based selective thermal emitter for infrared stealth technology. *Adv. Opt. Mater.* **6**, 1801006 (2018).

66 D. Qi, X. Wang, F. Chen, Y. Cheng, R. Gong, Metal-based graphical SiO_2/Ag/ZnS/Ag hetero-structure for visible-infrared compatible camouflage. *Materials* **11**, 1594 (2018).

67 H. Liu, C. Wang, G. Chen, Y. Liao, M. Mao, T. Cheng, A. Libanori, X. Xiao, X. Hu, K. Liu, J. Chen, Moisture assisted photo-engineered textiles for visible and self-adaptive infrared dual camouflage. *Nano Energy* **93**, 106855 (2022).

68 H. K. Woo, K. Zhou, S.-K. Kim, A. Manjarrez, M. J. Hoque, T. Y. Seong, L. Cai, Visibly transparent and infrared reflective coatings for personal thermal management and thermal camouflage. *Adv. Funct. Mater.* **32**, 2201432 (2022).

69 S. Dang, H. Ye, A visible-infrared-compatible camouflage photonic crystal with heat dissipation by radiation in 5–8 μm. *Cell Rep. Phys. Sci.* **2**, 100617 (2021).
